# A novel CAF-cancer cell crosstalk-related gene prognostic index based on machine learning: prognostic significance and prediction of therapeutic response in head and neck squamous cell carcinoma

**DOI:** 10.1186/s12967-024-05447-6

**Published:** 2024-07-09

**Authors:** Yuming Xu, Junda Li, Jinming Wang, Feilong Deng

**Affiliations:** Hospital of Stomatology, Guanghua School of Stomatology, Sun Yat-Sen University, Guangdong Provincial Key Laboratory of Stomatology, Guangzhou, 510055 China

**Keywords:** Head and neck squamous cell carcinoma, Immunotherapy, Machine learning, Prognosis, Biomarker, Cancer-associated fibroblasts

## Abstract

**Background:**

Cancer-associated fibroblast (CAF)-cancer cell crosstalk (CCCT) plays an important role in tumor microenvironment shaping and immunotherapy response. Current prognostic indexes are insufficient to accurately assess immunotherapy response in patients with head and neck squamous cell carcinoma (HNSCC). This study aimed to develop a CCCT-related gene prognostic index (CCRGPI) for assessing the prognosis and response to immune checkpoint inhibitor (ICI) therapy of HNSCC patients.

**Methods:**

Two cellular models, the fibroblast-cancer cell indirect coculture (FCICC) model, and the fibroblast-cancer cell organoid (FC-organoid) model, were constructed to visualize the crosstalk between fibroblasts and cancer cells. Based on a HNSCC scRNA-seq dataset, the R package CellChat was used to perform cell communication analysis to identify gene pairs involved in CCCT. Least absolute shrinkage and selection operator (LASSO) regression was then applied to further refine the selection of these gene pairs. The selected gene pairs were subsequently subjected to stepwise regression to develop CCRGPI. We further performed a comprehensive analysis to determine the molecular and immune characteristics, and prognosis associated with ICI therapy in different CCRGPI subgroups. Finally, the connectivity map (CMap) analysis and molecular docking were used to screen potential therapeutic drugs.

**Results:**

FCICC and FC-organoid models showed that cancer cells promoted the activation of fibroblasts into CAFs, that CAFs enhanced the invasion of cancer cells, and that CCCT was somewhat heterogeneous. The CCRGPI was developed based on 4 gene pairs: IGF1-IGF1R, LGALS9-CD44, SEMA5A-PLXNA1, and TNXB-SDC1. Furthermore, a high CCRGPI score was identified as an adverse prognostic factor for overall survival (OS). Additionally, a high CCRGPI was positively correlated with the activation of the P53 pathway, a high TP53 mutation rate, and decreased benefit from ICI therapy but was inversely associated with the abundance of various immune cells, such as CD4+ T cells, CD8+ T cells, and B cells. Moreover, Ganetespib was identified as a potential drug for HNSCC combination therapy.

**Conclusions:**

The CCRGPI is reliable for predicting the prognosis and immunotherapy response of HSNCC patients and may be useful for guiding the individualized treatment of HNSCC patients.

**Supplementary Information:**

The online version contains supplementary material available at 10.1186/s12967-024-05447-6.

## Background

In recent years, immune checkpoint inhibitor (ICI) therapy has shown significant survival benefits [1–4]. For example, therapeutic regimens targeting cytotoxic T-lymphocyte-associated protein 4 (CTLA4) and programmed death 1 (PD-1) increase tumor regression rates from less than 10% to nearly 50% in patients with advanced melanoma [2]. In head and neck squamous cell carcinoma (HNSCC), ICI therapy has been proven to be effective in treating patients with recurrence or metastasis [5–7]. Nevertheless, the response to ICI therapy in patients with HNSCC is limited to a relatively low percentage (13%–45%) [5, 8]. The construction of markers related to the prognosis of immunotherapy can provide personalized treatment options for HNSCC patients. Unfortunately, although some prognostic markers have been developed for HSNCC, these markers perform poorly in the HNSCC immunotherapy cohort (Fig. 8D). Therefore, effective prognostic and therapeutic indicators are still urgently needed.

Cancer-associated fibroblasts (CAFs) are the most abundant stromal cells in the tumor microenvironment (TME) and play a crucial role in tumor metastasis and immunosuppression through CAF-cancer cell crosstalk (CCCT) [9, 10]. However, it is still difficult to visualize the process of CCCT, which makes it more difficult to fully understand CCCT. Moreover, CAFs have been shown to impact the effectiveness of ICI therapy [11–13]. However, the functional state of CAFs and the patterns of CCCT may vary among different patients [9, 14], thus making it challenging to predict the correlation between CCCT and the efficacy of ICI therapy. Nevertheless, elucidating the ability of CCCT to predict the prognosis and response to ICI therapy in HNSCC patients is urgently needed because this will not only enhance our understanding of the role of CCCT in the development of HNSCC but can also be used to guide personalized treatment. Unfortunately, few studies have focused on the ability of CCCT to predict patient's prognosis and response to immunotherapy.

**Fig. 1:**
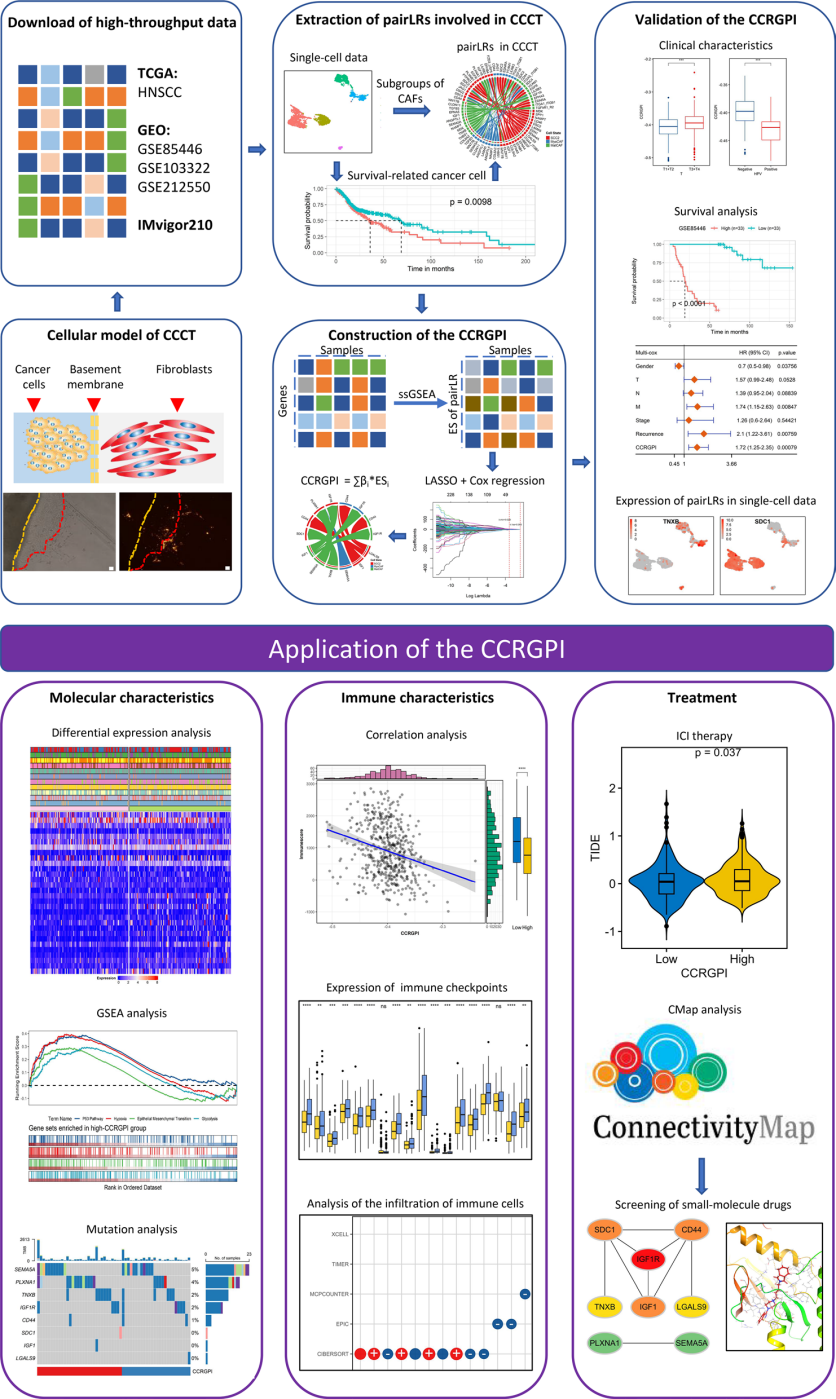
Flowchart of the entire research

In this study, we first constructed two cellular models to observe CCCT. Then, scRNA-seq data and bulk transcriptomic data of HNSCC patients were combined to develop a prognostic index for HNSCC that can distinguish the immune status and prognosis of patients receiving ICI therapy. Paired ligands and receptors (pairLRs) between CAFs and cancer cells were extracted using the scRNA-seq data of HNSCC, and the key pairLRs were identified through least absolute shrinkage and selection operator (LASSO) regression and Akaike information criterion (AIC)-based stepwise Cox regression to construct a CAF-cancer cell crosstalk-related gene prognostic index (CCRGPI). We then described the molecular and immune characteristics of the CCRGPI, studied its prognostic ability in patients receiving ICI therapy, and screened potential therapeutic drugs through the connectivity map (CMap) analysis and molecular docking (Fig. 1). The results showed that the CCRGPI can serve as a reliable biomarker to predict the prognosis and response to immunotherapy in HNSCC patients.

## Methods

### Construction of CCCT cellular models

**Fig. 2:**
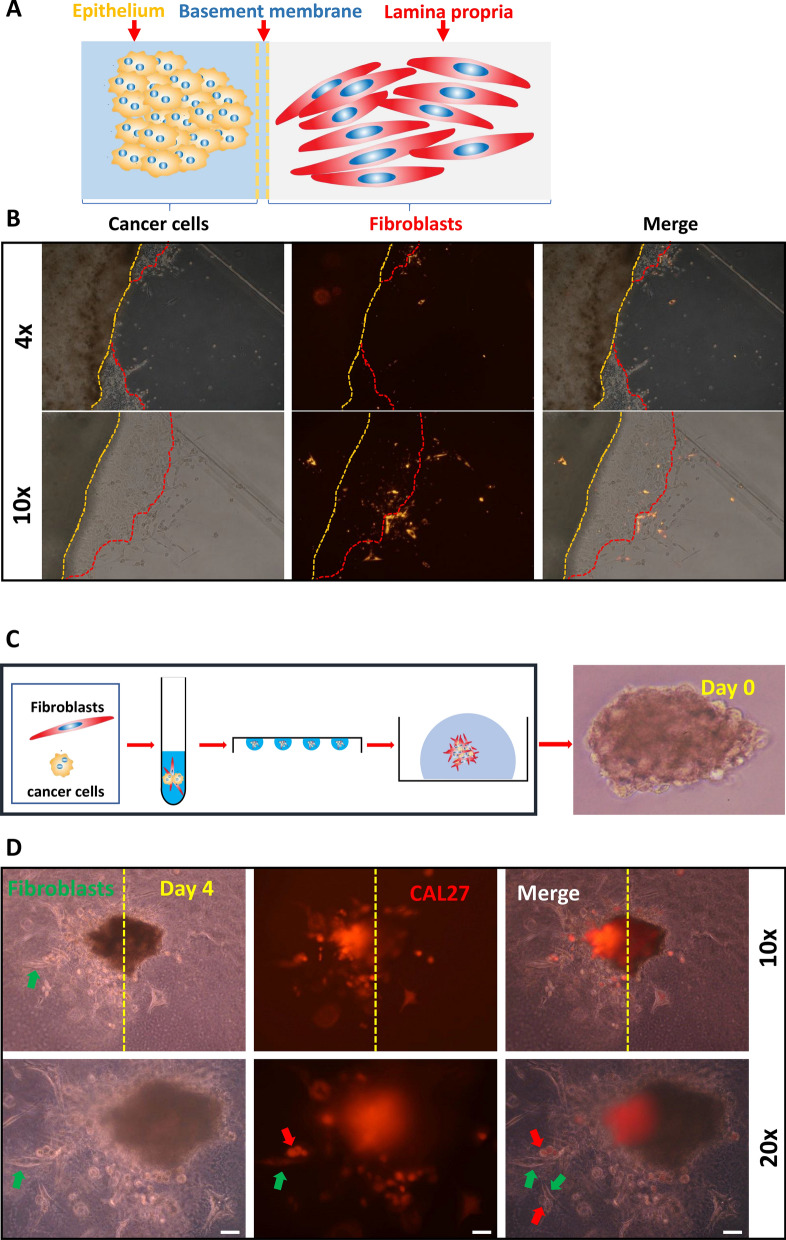
CCCT in cellular models. **A** Schematic diagram of the construction of the FCICC model. **B** Culture of the FCICC model. The red dotted line represents the leading edge of cancer cell invasion. The yellow dotted line represents the interface between Ca-mg and Fib-mg. **C** Schematic diagram of the construction of the FC-organoid model. **D** Culture of the FC-organoid model. The green arrow points to CAFs, and the red arrow points to CAL27 cells. *CAFs* cancer-associated fibroblasts, *Ca-mg* Matrigel containing cancer cells, *FCICC* fibroblast-cancer cell indirect coculture, *FC-organoid* fibroblast-cancer cell organoid, *Fib-mg* Matrigel containing fibroblasts

To visualize the crosstalk between fibroblasts and cancer cells, we constructed two cellular models. The first is the fibroblast-cancer cell indirect coculture model (FCICC). We inoculated fibroblasts and cancer cells into Matrigel (BD Biosciences, USA) at a density of 10^6^/100 µl, respectively. Next, 50 µl of Matrigel containing fibroblasts (Fib-mg) was added to the culture dish. After solidification, 50 µl of Matrigel containing cancer cells (Ca-mg) was inoculated on the surface of Fib-mg (Fig. 2A). After solidification, the cells were incubated in culture dishes supplemented with DMEM medium (HyClone, USA) and observed under a microscope every day.

Next, we constructed a fibroblast-cancer cell organoid model (FC-organoid). First, we prepared a cell suspension of fibroblasts and cancer cells at a ratio of 1:1 at a concentration of 10^5^ cells/ml and then added the cell suspension droplets onto the inverted 10CM dish lid, with 20 µl of suspension per droplet. The lid of the dish was then turned over, and the cells were cultured for 3 days to form cell spheroids. Next, the cell spheroids were injected into 50 µl of Matrigel, DMEM was added after solidification (Fig. 2C), and the cells were observed under a microscope every day. Fibroblasts and human oral squamous cell carcinoma cell line CAL27 were purchased from the Cell Bank and Stem Cell Bank, Chinese Academy of Science (Shanghai, China).

### Data sources and processing

RNA-seq data, mutation data, and clinicopathologic information for 494 HNSCC samples labeled “-01A” were downloaded from The Cancer Genome Atlas (TCGA) database (https://portal.gdc.cancer.gov/projects/TCGA-HNSC). RNA-seq data and survival information of 66 HNSCC samples (GSE85446), scRNA-seq data of 10 human papillomavirus (HPV)-negative HNSCC samples (GSE103322) [15], and immunotherapy data of HNSCC samples (GSE212550, anti-PD-1) were downloaded from the Gene Expression Omnibus (GEO) database (https://www.ncbi.nlm.nih.gov/geo). Missing data were replaced with NA. ScRNA-seq data was integrated and analyzed using the R packages “Harmony” [16] and “Seurat” [17], respectively. Genes that could only be detected in 5 or fewer cells, as well as low-quality cells with fewer than 2000 genes detected, were excluded from subsequent analysis. The top 2000 genes characterized by high variability were then screened by the FindVariableFeatures function in the “Seurat” package. Principal component analysis (PCA) was performed on the single-cell samples, and the top 30 principal components (PCs) were selected for subsequent analysis. The top 30 PCs were analyzed for overall dimensionality reduction of the samples using the UMAP algorithm [18].

### Extraction of pairLRs involved in CCCT

Based on the scRNA-seq data of 10 HNSCC samples, CAFs and cancer cells were divided into different subpopulations using the R package “Seurat” with a resolution of 0.1. The FindAllMarkers function was used to identify differentially expressed genes (DEGs) (p < 0.05, |logFC|> 1), and gene ontology (GO) analysis was performed on these genes using the R package “clusterProfiler” [19]. Then, the expression data of each subpopulation were uploaded to CIBERSORT (https://www.cibersort.stanford.edu) to determine the relative proportion of each subpopulation in HNSCC samples obtained from TCGA. Survival analysis was subsequently carried out to determine the survival-related subpopulations of cancer cells (p < 0.05, log-rank test). Finally, the R package “CellChat” [20] was used to screen the pairLRs between CAFs and survival-related subpopulations of cancer cells.

### Construction of the CCRGPI

First, the enrichment score (ES) of the piarLRs in the TCGA cohort was calculated using single sample gene set enrichment analysis (ssGSEA) provided in the “GSVA” package [21]. Based on the ES, all piarLRs were subjected to the LASSO regression to identify the key piarLRs when the partial likelihood deviance reached the minimum value. These key piarLRs were further transformed into binary variables and analyzed via the univariate Cox regression analysis. The piarLRs with p values less than 0.05 were subsequently subjected to the stepwise Cox regression based on the Akaike information criterion (AIC) to establish the CCRGPI. The model with the smallest AIC value was identified as the ultimate model. The CCRGPI was constructed according to the formula:

$${\text{CCRGPI}} = \sum\upbeta {\text{i}}*ES_{i} ,$$

where βi is the coefficient of each pairLR in the final model and $E{S}_{i}$ represents the ES of each pairLR. In subsequent analyses, the samples were subdivided into a high-CCRGPI group and a low-CCRGPI group based on the median CCRGPI or cutoff point calculated by the “survivalROC” package.

### Validation of the CCRGPI

To assess the reliability of the CCRGPI in predicting prognosis, we analyzed the associations of the CCRGPI with different clinicopathological factors using Wilcoxon tests. In addition, Kaplan‒Meier (KM) survival curves were plotted for the TCGA dataset and the GEO dataset (GSE85446) using the R package “survminer”. To explore the sensitivity and specificity of the CCRGPI, we carried out a time-dependent area under the receiver operating characteristic curve (AUC) analysis with the R package “riskRegression” [22]. Then, we performed univariate and multivariate Cox regression analyses of the CCRGPI and clinicopathologic factors in the TCGA cohort using the R package “survival”.

### Analysis of molecular characteristics in different CCRGPI subgroups

First, differential expression analysis was performed on the two CCRGPI subgroups using the “edgeR” [23] package to obtain all DEGs. Genes with p < 0.05 and |logFC|> 1 were considered significantly DEGs. Then, based on the HALLMARK and GO gene sets downloaded from MSigDB (https://www.gsea-msigdb.org/gsea/msigdb), gene set enrichment analysis (GSEA) was performed on the DEGs using the GSEA function in the GSVA package to screen the signaling pathways in which the DEGs were involved (p < 0.05). Subsequently, the “Maftools” package [24] was used to analyze gene mutations and tumor mutation burden (TMB) in the two CCRGPI subgroups.

### Analysis of immune characteristics in different CCRGPI subgroups

The immunescores and stromalscores were calculated using the R package “estimate” [25] and were subsequently used to analyze correlations with CCRGPI. The immune-related gene sets HCK, IgG, Interferon, LCK, MHC_I, MHC_II, and STAT1 were obtained from Zhang [26]. Then, the ES of these gene sets was calculated by the ssGSEA algorithm. The relative proportions of immune cells in HNSCC samples based on the XCELL, TIMER, MCPCOUNTER, EPIC, and CIBERSORT algorithms were downloaded from TIMER (RRID:SCR_018737) (http://timer.comp-genomics.org). Subsequently, we used the “limma” package [27] to analyze the differences in the relative proportions of immune cells. Finally, differences in the expression of 46 immune checkpoints [28] in the two CCRGPI subgroups were examined and visualized using the R package “ggpubr”.

### Prediction of therapeutic sensitivity in different CCRGPI subgroups

We inferred patients’ potential response to immunotherapy through the tumor immune dysfunction and exclusion (TIDE) score and the immunophenoscore (IPS). Generally, the lower the TIDE score and the higher the IPS, the better the response to immunotherapy. The TIDE score was calculated on the TIDE website (http://tide.dfci.harvard.edu), and the IPS was downloaded from The Cancer Immunome Database (TCIA) (https://tcia.at/home). Then, immunotherapy data from patients with HNSCC (GSE212550, anti-PD-1, n = 20) and urothelial cancer (IMvigor210, anti-PD-L1, n = 298) [29] were used to investigate the ability of the CCRGPI to predict the prognosis of patients receiving ICI therapy. Finally, we used the “oncoPredict” [30] package to extrapolate the 50% inhibitory concentration (IC50) values of 198 chemotherapy/targeted therapy drugs in the two CCRGPI subgroups to investigate the predictive ability of the CCRGPI to predict the response to immunotherapy, chemotherapy, and targeted therapy.

### Screening of small-molecule drugs and key target

To screen for small-molecule drugs against CCRGPI, we selected the 80 upregulated genes with the largest logFC and the 80 downregulated genes with the smallest logFC from DEGs for connectivity map (CMap) analysis (https://clue.io/query) [31]. Drugs with a mean connective score < − 0.4 and p < 0.05 were retained, and the chemical structures of these drugs were downloaded from the PubChem database (https://pubchem.ncbi.nlm.nih.gov) [32].

To identify the key target of CCRGPI, we constructed a protein–protein interaction (PPI) network by imputing 8 proteins contained in CCRGPI into the STRING database (https://string-db.org/) [33]. Then, the maximum neighborhood component centrality, neighborhood component centrality, and clustering coefficient algorithms were performed to mine the key target using the cytoHubba plug-in contained in Cytoscape (RRID: SCR_003032).

### Molecular docking

Based on the best available resolution, we downloaded the crystal structure of the key target from the Protein Data Bank (PDB) database (http://www.rcsb.org) [34], and this crystal structure was poured along with the 3D structures of drugs derived from CMap analysis into the Schrödinger software suite to perform molecular docking. First, the protein crystal structure was optimized by the Protein Preparation Wizard module. Next, the LigPrep module was used for ligand preparation. Subsequently, the active site of the protein is identified and the Receptor Grid Generation tool is used to create a grid at the site, which will serve as a space for ligand exploration during subsequent docking. Then, the Glide module is used to perform ligand searches and docking within the generated grid. Upon completion of docking, the binding affinity of the ligand to the target protein was evaluated based on the Glide score. Lower Glide scores usually indicate superior binding possibilities.

### Statistical analysis

For continuous variables, the Wilcoxon test was utilized for the comparisons between two groups. For categorical variables, the chi-square test was used to calculate the significance of differences, and KM (log-rank test) or Cox regression analysis was used for survival analysis. In the correlation analysis between the CCRGPI score and other variables, the Spearman method was used to calculate correlation coefficients and p values. In this research, p values < 0.05 were considered significant.

## Results

### CCCT in cellular models

First, we simulated the process of cancer cells breaking through the basement membrane from the epithelium into the lamina propria using the FCICC model (Fig. 2A). Interestingly, cancer cells advanced into the lamina propria in a jagged manner, and a large number of fibroblasts were activated in Fi-mg invaded by cancer cells and aggregated at the leading edge of the cancer cell invasion. In contrast, few fibroblasts were activated in Fi-mg not invaded by cancer cells (Fig. 2B). This finding confirms the critical role of cancer cells in promoting the transformation of fibroblasts into CAFs. To explore the effect of CAFs on cancer cells, we constructed an FC-organoid model (Fig. 2C). Notably, CAFs and cancer cells grew asymmetrically in the FC-organoid model (Fig. 2D), with more cancer cells on the side where CAFs were active. In addition, CAFs grew outward more rapidly than cancer cells, and there was concomitant exogenesis between them. These results suggested that CCCT has a certain degree of heterogeneity, which may be caused by differences at the cellular and molecular levels. Therefore, we explored the heterogeneity of CAFs and cancer cells using scRNA-seq data from HNSCC patients in subsequent studies.

### PairLRs involved in CCCT

**Fig. 3:**
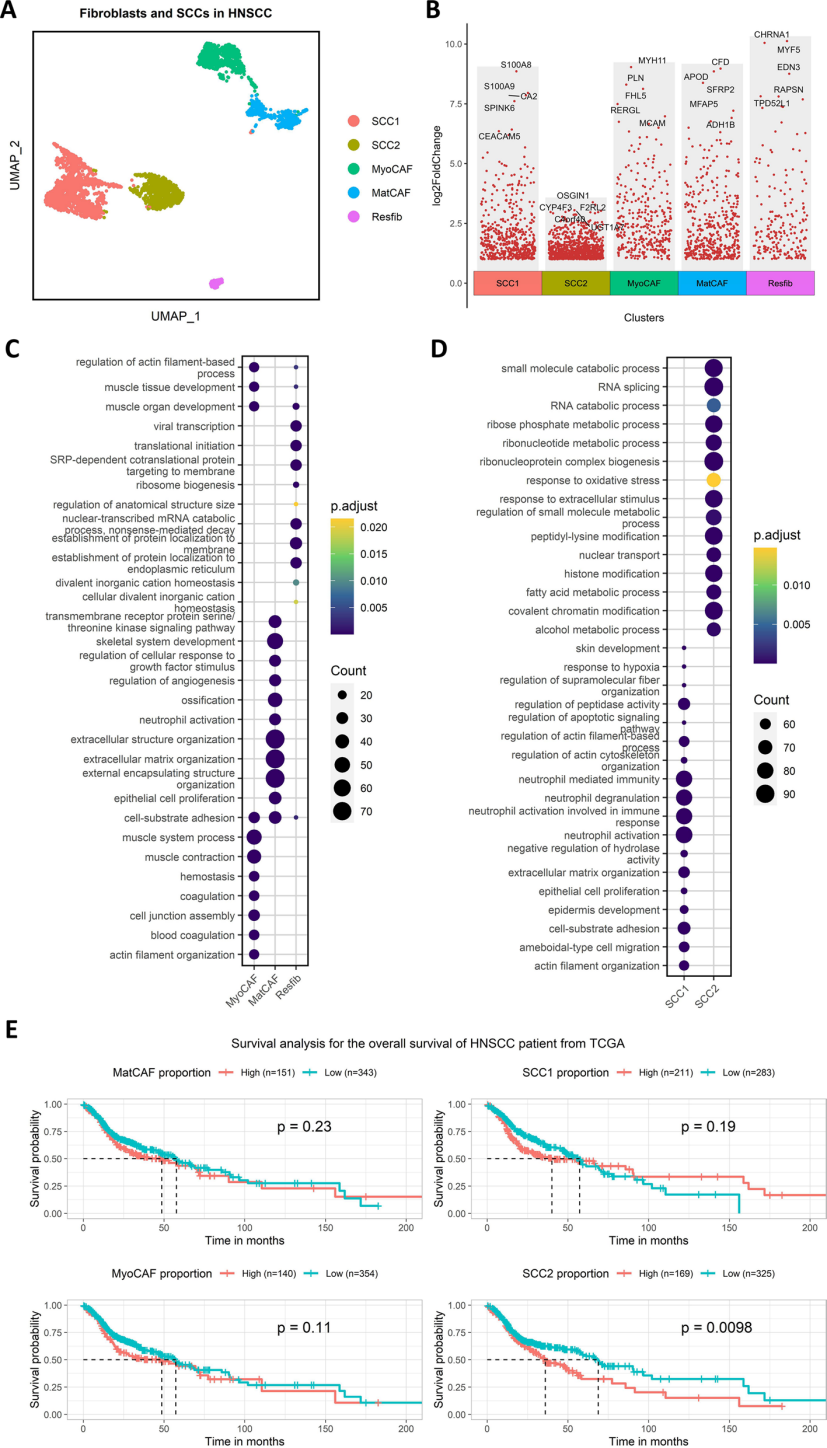
Characteristics of single-cell subgroups. **A** Dimensionality reduction analysis of the scRNA-seq data. **B** Highly expressed genes in single-cell subgroups. **C**, **D** GO analysis. **E** Kaplan‒Meier survival analysis of the single-cell subgroups in the TCGA cohort. *GO* Gene Ontology, *TCGA* The Cancer Genome Atlas

After quality control and integration of the scRNA-seq data (Additional file 1: Figure S1A, 1B), cancer cells from 10 HSNCC samples were classified into two squamous cell carcinoma (SCC) subgroups (SCC1, SCC2), and CAFs were divided into three subgroups, namely, myofibroblast (MyoCAF), matrix CAF (MatCAF), and resting fibroblast (Resfib). (Fig. 3A), with each subgroup exhibiting its own highly expressed genes (Fig. 3B and Additional file 1: Figure S1D). The expression of CAFs markers varied across the three CAFs subgroups, with MyoCAF exhibiting high levels of ACTA2, MatCAF showing elevated expression of FAP, COL1A1, and DCN, and Resfib expressing the mesenchymal marker VIM (Additional file 1: Figure S1C). Puram [15] categorized Resfib as inactivated fibroblasts; hence, we excluded Resfib from subsequent analyses. The results from the GO analysis indicated that genes overexpressed in MyoCAF were predominantly enriched in pathways related to myogenesis and contraction, while those overexpressed in MatCAF were mainly enriched in extracellular matrix-associated pathways; for cancer cells, genes highly expressed in SCC1 were chiefly enriched in pathways related to neutrophils, whereas those in SCC2 were mainly enriched in metabolic and stress-related pathways (Fig. 3C and D). Notably, survival analysis revealed that HNSCC patients with a higher proportion of SCC2 cells had a poorer prognosis (Fig. 3E). Therefore, we extracted only pairLRs between MyoCAF, MatCAF, and SCC2 cells for subsequent studies (Additional file 1: Figure S2).

### Construction of the CCRGPI

**Fig. 4:**
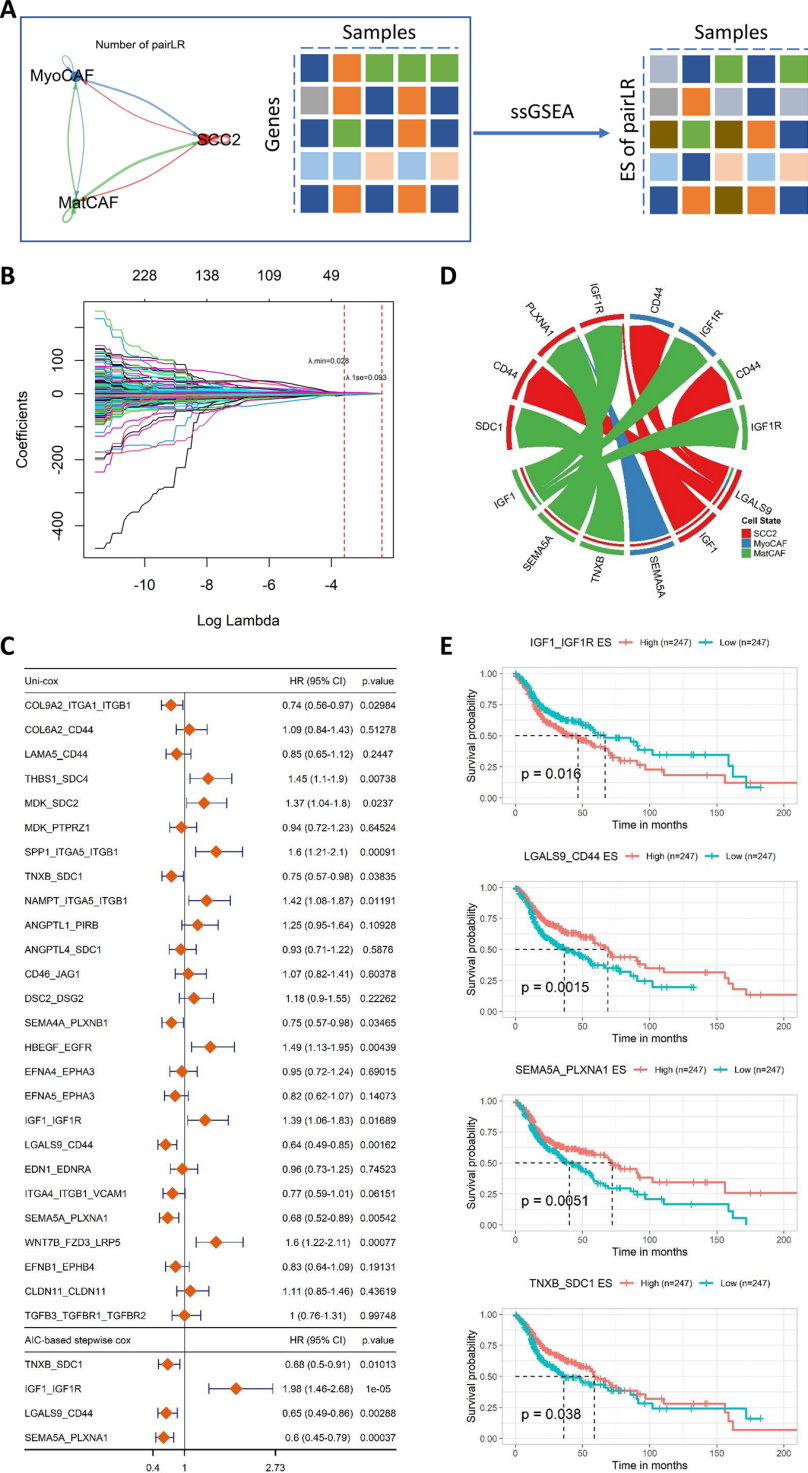
Construction of the CCRGPI. **A** Schematic diagram of CCRGPI construction. **B** LASSO regression analysis of the pairLRs. **C** Univariate Cox analysis of the pairLRs and stepwise Cox regression of the pairLRs significant in the univariate Cox analysis (p < 0.05). **D** PairLRs contained in the CCRGPI. **E** Kaplan‒Meier survival analysis of the pairLRs contained in the CCRGPI. *CCRGPI* cancer-associated fibroblast (CAF)-cancer cell crosstalk-related gene prognostic index, *LASSO* least absolute shrinkage and selection operator, *pairLRs* paired ligands and receptors

To identify the key pairLRs for the construction of the CCRGPI, we first employed the ssGSEA algorithm to calculate the ES of pairLRs in HNSCC samples from TCGA (Fig. 4A). Subsequently, these pairLRs were subjected to LASSO regression analysis (Fig. 4B), from which 26 critical pairLRs were selected (Additional file 1: Figure S3A). These pairLRs were then used to perform univariate Cox regression analysis, and 10 pairLRs were ultimately selected (Fig. 4C). Next, these 10 pairLRs were input into a stepwise Cox regression analysis, ultimately retaining 4 pairLRs (Fig. 4C, D). Based on the 4 pairLRs, we constructed a model using the following formula: CCRGPI = (0.682) * IGF1_IGF1R + (− 0.425)  * LGALS9_CD44 + (− 0.518)*SEMA5A_PLXNA1 + (− 0.392)  * TNXB_SDC1. Consistent with the formula, HNSCC patients with high IGF1_IGF1R ES had a worse prognosis, which was reversed for LGALS9_CD44, SEMA5A_PLXNA1, and TNXB_SDC1 (Fig. 4E).

### Validation of the CCRGPI

**Fig. 5:**
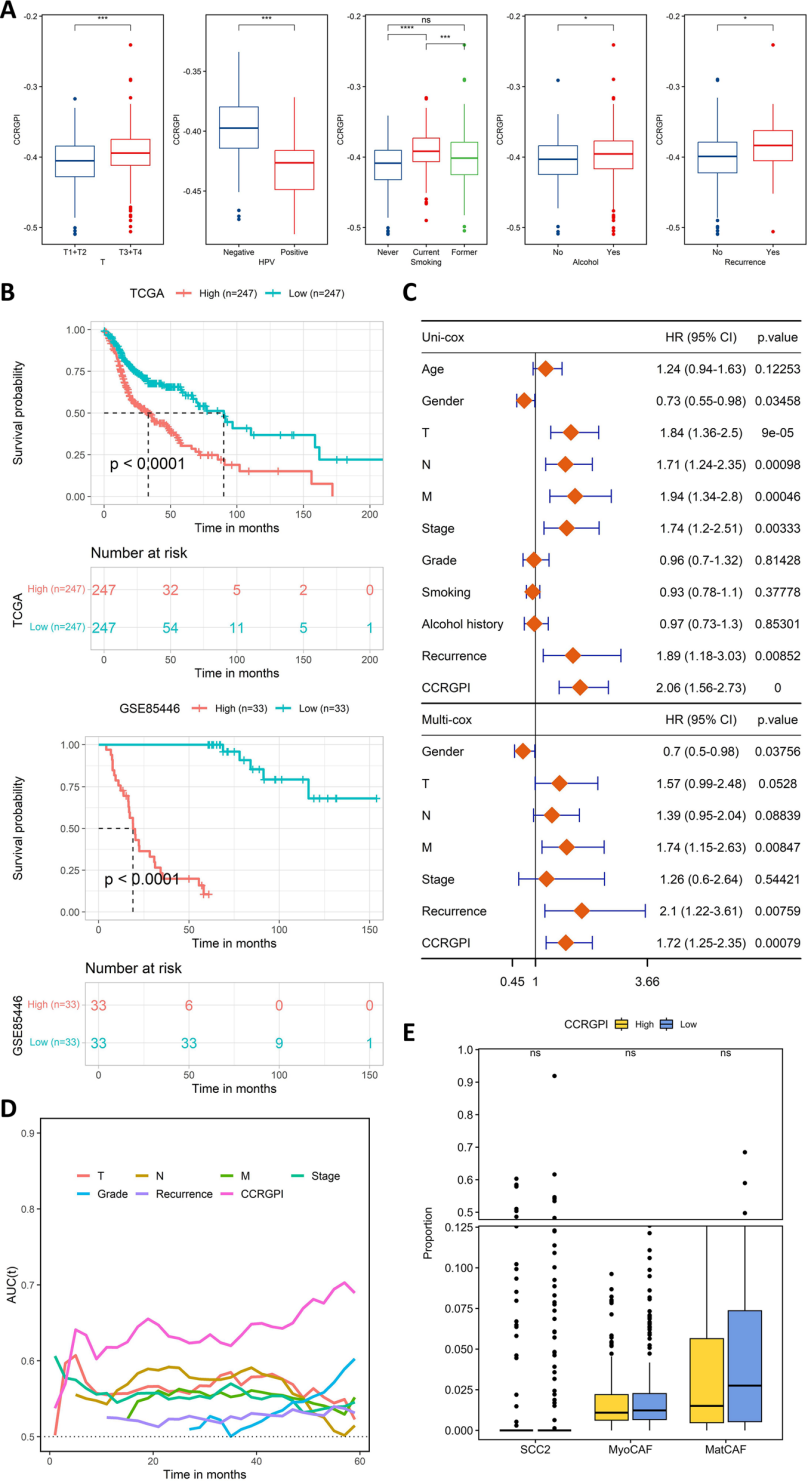
Validation of the CCRGPI. **A** CCRGPI in samples with different clinicopathologic factors (ns: not significant, *p < 0.05, ***p < 0.001, ****p < 0.0001). **B** Kaplan‒Meier survival analysis of the CCRGPI subgroups in the TCGA and GEO cohorts. **C** Univariate Cox analysis of clinicopathologic factors and CCRGPI and multivariate Cox analysis of factors significant in univariate Cox analysis (p < 0.05). **D** Time-dependent AUC analysis of clinicopathologic factors and CCRGPI. **E** Proportion of MatCAF, MyoCAF, and SCC2 cells in the TCGA cohort (ns: not significant). *AUC* area under the receiver operating characteristic curve, *CCRGPI* cancer-associated fibroblast (CAF)-cancer cell crosstalk-related gene prognostic index, *GEO* Gene Expression Omnibus, *MatCAF* matrix CAF, *MyoCAF* myofibroblast, *SCC2* squamous cell carcinoma 2, *TCGA* The Cancer Genome Atlas

We first studied the distribution of the CCRGPI in HNSCC patients with different clinical characteristics. The results showed that patients with a history of smoking, drinking, recurrence, and advanced T stage had a greater CCRGPI, but HPV-positive patients had a lower CCRGPI (Fig. 5A). Consistently, the proportion of patients with a history of smoking, drinking, recurrence, and advanced T stage was higher in the high-CCRGPI group, but the proportion of HPV-positive patients was lower (Additional file 1: Figure S3C). As shown in Additional file 1: Figure S3B, the HPV-positive group had significantly lower content of MatCAF. It has been indicated that HPV-positive HSNCC patients [35, 36] and those with low stroma abundance [37, 38] have a better prognosis. MatCAF is an important cell for stroma production, so a low level of MatCAF may be one of the reasons why HPV-positive patients have a better prognosis. Although the content of SCC2 was higher in the HPV-positive group, the reduction in MatCAF may lead to a lower CCRGPI (Fig. 5A), which in turn led to an overall effect of better prognosis.

KM curves (log-rank test) and univariate Cox analysis showed that patients with higher CCRGPI had worse OS (Figs. 5B and C). In multivariable Cox regression analysis, CCRGPI was confirmed as an independent risk factor for OS in the TCGA dataset after adjusting factors such as gender, T stage, N stage, M stage, clinical stage, and recurrence (Fig. 5C). Moreover, time-dependent AUC analysis suggested that the CCRGPI has considerable value in predicting the OS of patients with HSNCC in the TCGA cohort (Fig. 5D). Notably, there were no significant differences in the proportions of SCC2, MyoCAF, and MatCAF between the two CCRGPI subgroups (Fig. 5E), indicating that the predictive power of CCRGPI was independent of the content of these 3 cell types. ScRNA-seq data analysis revealed that 8 genes contained in the CCRGPI were differentially expressed in SCC2, MyoCAF, and MatCAF and exhibited extensive crosstalk between these cells (Fig. 4D and Additional file 1: Figure S4), which further confirmed the reliability of CCRGPI and its role in characterizing CCCT.

### Molecular characteristics in different CCRGPI subgroups

**Fig. 6:**
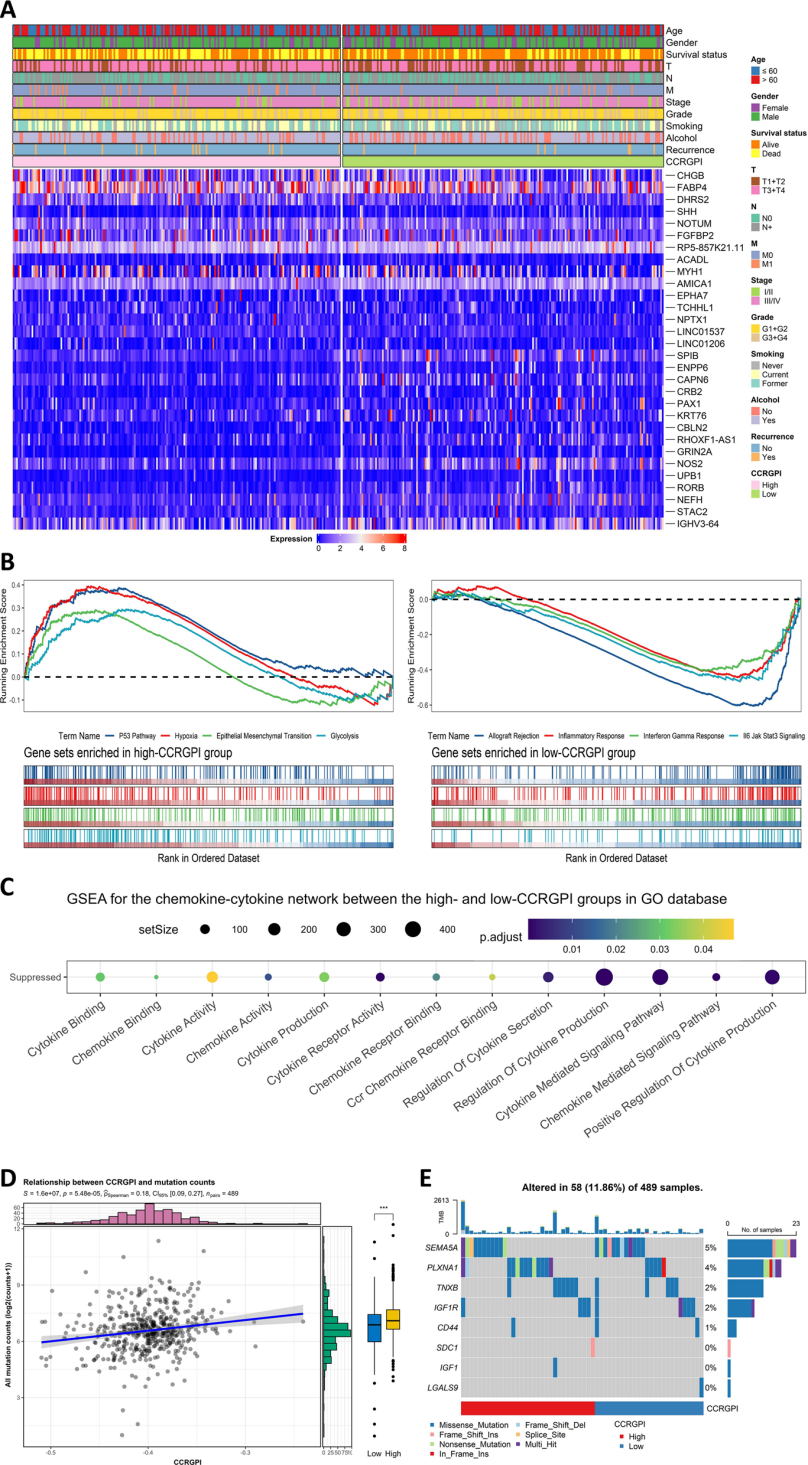
Molecular characteristics in different CCRGPI subgroups. **A** DEGs and clinical features in different CCRGPI subgroups. **B** GSEA analysis on the DEGs based on the HALLMARK gene sets (p < 0.05). **C** GSEA analysis on the DEGs based on chemokine-cytokine network in the GO database (p < 0.05). **D** Correlations between the gene mutations and CCRGPI. **E** Mutations of genes contained in the CCRGPI. *CCRGPI* cancer-associated fibroblast (CAF)-cancer cell crosstalk-related gene prognostic index, *DEGs* differentially expressed genes, *GO* Gene Ontology, *GSEA* gene set enrichment analysis

In the differential expression analysis, 48 genes were upregulated in the high-CCRGPI group compared to the low-CCRGPI group, which was much lower than the 279 downregulated genes (Fig. 6A and Additional file 1: Figure S5A). Even so, GSEA analysis based on the HALLMARK gene sets showed that up to 13 signaling pathways were activated in the high-CCRGPI group, such as the P53 Pathway, hypoxia, epithelial-mesenchymal transition, and glycolysis, while only allograft rejection, inflammatory response, interferon gamma response, and IL6 Jak Stat3 signaling were suppressed (Fig. 6B and Additional file 1: Figure S5B), which suggested that the high-CIRGS group may have a higher malignancy grade. Additionally, GSEA analysis based on the chemokine-cytokine network showed that the production, secretion, binding, and activation of chemokines and cytokines were all inhibited in the high-CCRGPI group (Fig. 6C). Chemokines and cytokines, such as IFN-γ [39], CXCL9 [40], and CCL20 [41], are related to the recruitment of CD4+ and CD8+ T cells, so the lack of chemokines and cytokines may lead to impaired recruitment of immune cells, thereby causing an immunosuppressive tumor microenvironment.

The gene mutation is an important driver of TME heterogeneity. For example, the TP53 gene, which is the most frequently mutated gene in human cancer, can exert tumor-suppressive effects through the regulation of immunity, and its mutation will alter the immune microenvironment and promote the development of cancer [42]. Therefore, we calculated the TMB of HNSCC samples. Correlation analysis revealed a positive correlation between the TMB and CCRGPI, with the high-CCRGPI group exhibiting a greater TMB (Fig. 6D and Additional file 1: Figure S5G). Next, we extracted the top 10 genes with the highest mutation frequency and those with significant mutation differences (p < 0.01). TP53 was identified as the most frequently mutated gene, with a notably higher mutation frequency in the high-CCRGPI group (73%) than that in the low-CCRGPI group (62%) (Additional file 1: Figure S5C). Additionally, the mutation frequency of 19 genes with significant mutational differences was higher in the high-CCRGPI group (Additional file 1: Figure S5D), and these gene mutations demonstrated a significant co-occurrence (Additional file 1: Figure S5E). Furthermore, the mutation rate of the 8 genes included in the CCRGPI was low, with no significant differences between the two CCRGPI subgroups (Fig. 6E). However, there was a significant co-occurrence of TNXB and IGF1 (Additional file 1: Figure S5F).

### Immune characteristics in different CCRGPI subgroups

**Fig. 7:**
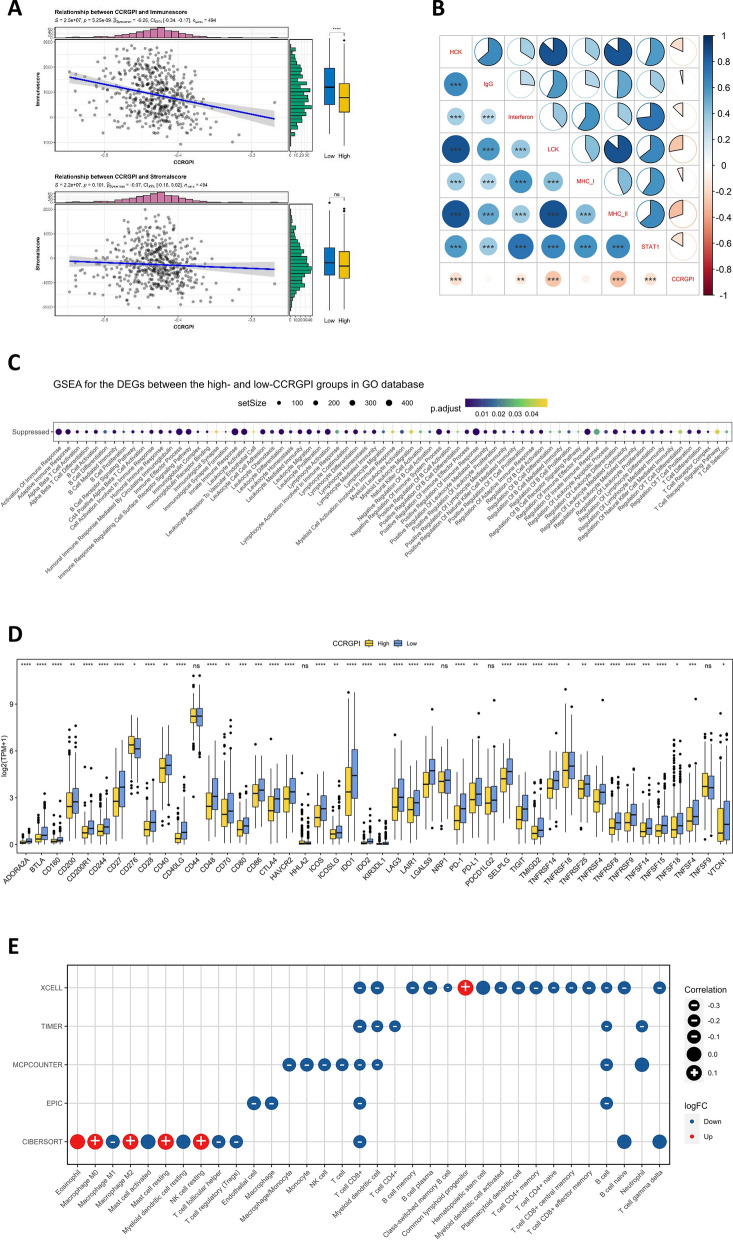
Immune characteristics in different CCRGPI subgroups. **A** Correlation between the immunescore, stromalscore, and CCRGPI (ns: not significant, ****p < 0.0001). **B** Correlation between the ES of immune-related gene sets and CCRGPI (**p < 0.01, ***p < 0.001). **C** GSEA analysis on the DEGs based on immune-related gene sets in the GO database (p < 0.05). **D** Expression of 46 immune checkpoints in different CCRGPI subgroups (ns: not significant, *p < 0.05, **p < 0.01, ***p < 0.001, ****p < 0.0001). **E** Proportion of immune cells in the TCGA cohort based on the XCELL, TIMER, MCPCOUNTER, EPIC, and CIBERSORT algorithms. *CCRGPI* cancer-associated fibroblast (CAF)-cancer cell crosstalk-related gene prognostic index, *ES* enrichment score, *GO* Gene Ontology, *GSEA* gene set enrichment analysis, *TCGA* The Cancer Genome Atlas

Initially, the R package “estimate” was used to calculate the immunescore and stromalscore for HNSCC samples. CCRGPI was negatively correlated with the immunescore, and the high-CCRGPI group had a significantly lower immunescore than the low-CCRGPI group (Fig. 7A). However, there was no significant difference in the stromalscore between the two CCRGPI subgroups (Fig. 7A). Considering that CAFs are the most abundant stromal cells within the TME and that the subpopulations of CAFs did not significantly differ between the two CCRGPI subgroups, the absence of difference in stromalscore is reasonable and further substantiates the reliability of CCRGPI.

Subsequently, we investigated the relationships between CCRGPI and seven gene sets representing different inflammatory and immune responses. The results revealed that the ES of HCK, Interferon, LCK, MHC_II, and STAT1 gene sets were all negatively correlated with CCRGPI (Fig. 7B). Additionally, GSEA analysis based on the GO database indicated that immune-related pathways were all suppressed in the high-CCRGPI group compared to the low-CCRGPI group (Fig. 7C). Similarly, except for CD276, nearly all 46 immune checkpoint genes were downregulated in the high-CCRGPI group (Fig. 7D). To analyze the composition of immune cells within different CCRGPI subgroups, we also examined the distribution of infiltrating immune cells predicted by algorithms such as XCELL, TIMER, MCPCOUNTER, EPIC, and CIBERSORT. Our findings demonstrated that most immune cells, such as M1 macrophages, regulatory T cells (CD4+), cytotoxic T cells (CD8+), and dendritic cells, were reduced in the high-CCRGPI group and were negatively correlated with the CCRGPI. However, the infiltration of M0 macrophages, M2 macrophages, resting mast cells, resting NK cells, and common lymphoid progenitors was more abundant in the high-CCRGPI group and positively correlated with the CCRGPI (Fig. 7E). These results suggest that samples in the high-CCRGPI group were in an immunosuppressed state and may be insensitive to immunotherapy.

### Immune characteristics in different HPV subgroups

HPV-positive and HPV-negative HNSCC patients have different immune response patterns [36]. Therefore, we next investigated the immune characteristics in different HPV subgroups. As shown in Additional file 1: Figure S6A, in both HPV subgroups, CCRGPI was negatively correlated with the immunescore, and the high-CCRGPI group had a significantly lower immunescore than the low-CCRGPI group in the HPV-negative group. Moreover, the CCRGPI was negatively correlated with various immune-related gene sets, such as HCK and MHC_II in the HPV-negative group, and LCK, MHC_I, MHC_II, and STAT1 in the HPV-positive group (Additional file 1: Figure S6B). Additionally, in both HPV groups, all differentially expressed immune checkpoint genes were downregulated in the high-CCRGPI group (Additional file 1: Figure S6C). Similarly, almost all immune cells with differential content were reduced in the high-CCRGPI group, such as B cells, CD4+ T cells, and CD8+ T cells (Additional file 1: Figure S6D). Unfortunately, the HNSCC immunotherapy data used in this study did not contain information on HPV infection, thus we were unable to assess the performance of CCRGPI in predicting immunotherapy response in both HPV subgroups. Overall, whether in the HPV-negative or HPV-positive group, the high-CCRGPI group had lower immunogenicity, suggesting that HNSCC patients with a high CCRGPI are more difficult to benefit from ICI regardless of HPV infection. These results further confirmed the reliability of the CCRGPI.

### Therapeutic sensitivity in different CCRGPI subgroups

**Fig. 8:**
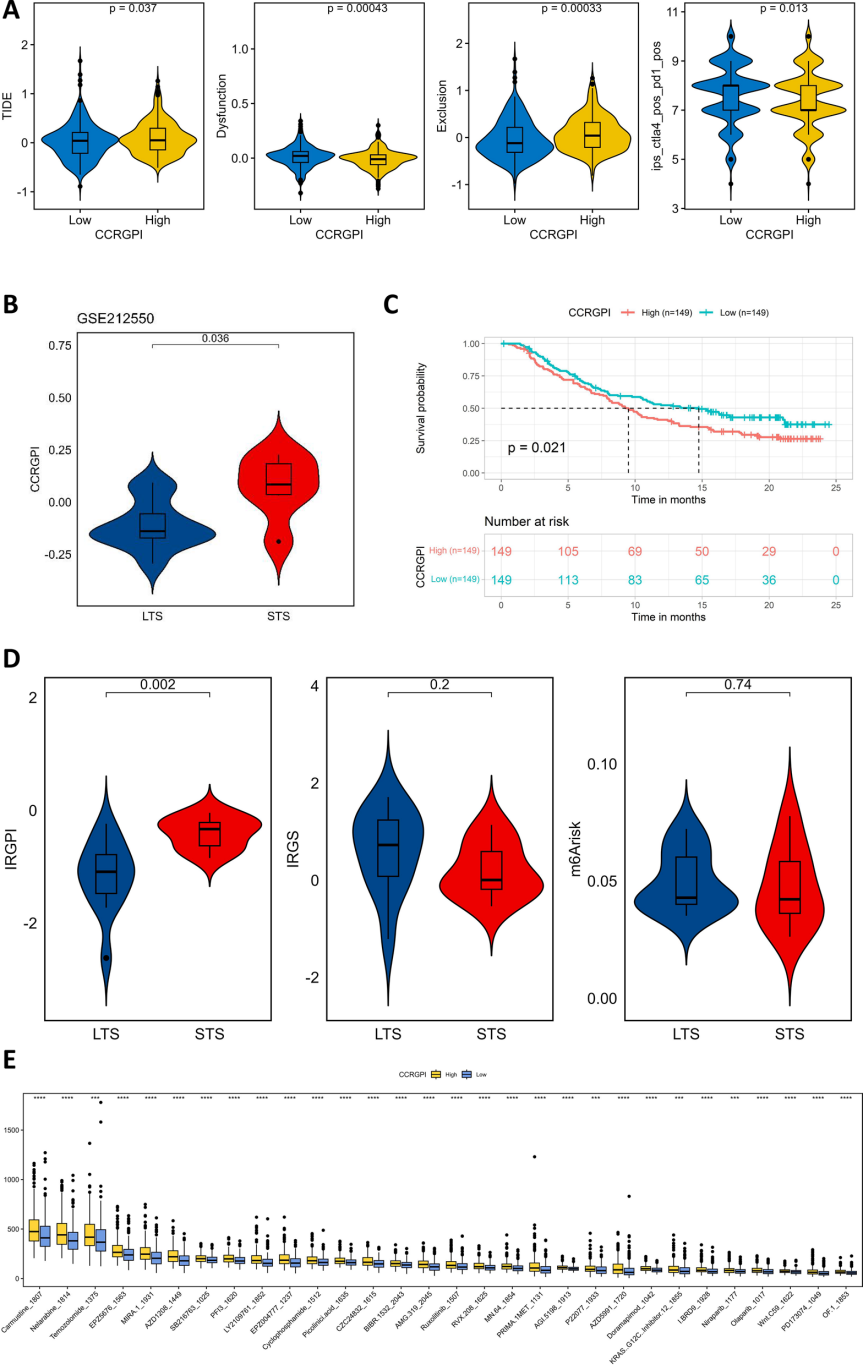
Therapeutic sensitivity in different CCRGPI subgroups. **A** TIDE, T-cell dysfunction, T-cell exclusion score, and IPS in different CCRGPI subgroups. **B** Comparison of the CCRGPI between the STS and LTS groups in the GSE212550 dataset. **C** Kaplan‒Meier survival analysis of different CCRGPI subgroups in the IMvigor210 dataset. **D** Comparison of the IRGPI, IRGS, and m6Arisk between the STS and LTS groups in the GSE212550 dataset. **E** Predicted IC50 values of 30 drugs in different CCRGPI subgroups (***p < 0.001, ****p < 0.0001). *CCRGPI* cancer-associated fibroblast (CAF)-cancer cell crosstalk-related gene prognostic index, *IC50* 50% inhibitory concentration, *IPS* immunophenoscore, *IRGPI* immune-related gene prognostic index, *IRGS* immune-related gene signature, *LTS* long-term survivors, *m6Arisk* m6A-based risk score, *STS* short-term survivors, *TIDE* tumor immune dysfunction and exclusion

We further explored the capability of the CCRGPI to predict the response to immunotherapy. First, we inferred patients' response to immunotherapy by the TIDE score and IPS. In general, the lower the TIDE score and the higher the IPS, the better the patient's response to immunotherapy. The results showed that the TIDE and T-cell exclusion scores of the high-CCRGPI group were higher than those of the low-CCRGPI subgroup, but the T-cell dysfunction score and IPS were lower (Fig. 8A). In addition, we investigated the ability of the CCRGPI to predict the prognosis of patients receiving ICI therapy using the immunotherapy datasets of HNSCC (GSE212550, anti-PD-1) and uroepithelial cancer (IMvigor210, anti-PD-L1). We found that patients with a high CCRGPI had a worse prognosis than those with a low CCRGPI (Figs. 8B and C). These results imply that HNSCC patients with a high CCRGPI are less likely to benefit from ICI therapy than those with a low CCRGPI. Interestingly, the results of the analysis of the immune-related gene prognostic index (IRGPI) [43], immune-related gene signature (IRGS) [44], and m6A-based risk score (m6Arisk) [45] in the GSE212550 dataset were contrary to the trends in the original articles (IRGPI, IRGS) or were not significant (m6Arisk) (Fig. 8D). Finally, we extrapolated the IC50 values of 198 chemotherapy/targeted therapy drugs in the two CCRGPI subgroups. The results showed that, among the 83 drugs with statistical significance, the samples in the high-CCRGPI group had higher IC50 values, that is, worse drug sensitivity (Fig. 8E and Additional file 2: Table S1). Overall, we suggest that the CCRGPI has considerable predictive ability for immunotherapy and even chemotherapy.

### Screening of small-molecule drugs and molecular docking

By using CMap analysis, we screened 20 potential small-molecule drugs against CCRGPI, such as Agomelatine, Prochlorperazine, PD-173074, and Ganetespib (Additional file 1: Figure S7A). To further identify the key target of CCRGPI, we built a PPI network consisting of 8 proteins contained in the CCRGPI. In addition, three algorithms were used to identify key targets. The results showed that IGF1R ranked first among all three algorithms and was therefore considered the key target (Additional file 1: Figure S7B). The molecular docking technique is an effective and rapid method for screening compounds and assessing the binding stability of a compound to a target by calculating the free binding energy. The top 15 compounds that bind well to IGF1R are listed in Additional file 2: Table S2. In particular, the binding energies of Perospirone and Ganetespib to IGF1R were lower than that of the native ligand (PubChem CID: 137349240), which implies that these two compounds have a greater affinity for IGF1R, suggesting that Perospirone and Ganetespib can be used for combination therapy of HNSCC. However, Perospirone is an atypical antipsychotic, so it was excluded [46]. A three-dimensional diagram of the interaction between Ganetespib and IGF1R is displayed in Additional file 1: Figure S7C.

## Discussion

In recent years, immunotherapy has shown good promise for solid tumors [1–4]. In particular, ICI therapy has shown positive results in the treatment of patients with HNSCC [5–7]. However, only a small number of patients are currently able to benefit significantly from this treatment. Therefore, the development of biomarkers that can accurately predict the prognosis of immunotherapy is of particular importance.

Numerous studies have developed prediction models for HNSCC immunotherapy based on immune-related genes or other gene sets, such as the IRGPI [43], IRGS [44], and m6Arisk [45]. However, these models were not validated in the HNSCC immunotherapy cohort. In this study, to validate the predictive ability of the above three biomarkers, we applied them to the HNSCC immunotherapy cohort containing the long-term survivors (LTS) and short-term survivors (STS) groups. Unfortunately, the results were contrary to the trend of the original article or were not significant. One of the reasons for the poor performance of these prediction models may be that they only pay attention to a single gene and ignore the heterogeneity of the TME when constructing models. It should be noted that the success of immunotherapy depends on multiple anti-immunotherapeutic mechanisms mediated by multifaceted crosstalk between stromal, epithelial, and immune cells within the TME [47].

Emerging evidence suggests a correlation between poor tumor immunotherapy response and CAFs. CAFs are the most abundant mesenchymal cells in the TME and one of the most important factors affecting the outcome of ICI therapy [11–13]. By remodeling the extracellular matrix and interacting with tumor cells and immune cells, CAFs can promote the formation of an immunosuppressive microenvironment and induce resistance to radiotherapy and chemotherapy [14, 48, 49]. In addition, different subsets of CAFs have different molecular features and functional properties and exhibit a certain degree of heterogeneity [50], which further complicates their effects on the TME and immune responses. Therefore, the development of biomarkers based on the crosstalk of CAFs with other cells in the TME is promising and may help to develop more effective therapeutic strategies and improve the success rate of immunotherapy.

Although the crosstalk between CAFs and immune cells has a more direct impact on the immune environment of the TME, the impact of the CCCT on the immune status of tumor should not be overlooked [48]. We observed in the FCICC and FC-organoid cellular models that the activation of CAFs was directly affected by cancer cells. Moreover, activated CAFs grew outward more rapidly than cancer cells, and there was concomitant outward growth with cancer cells. We hypothesized that CCCT may play a key role in the initiation stage of cancer development. Through CCCT, a large number of normal fibroblasts are transformed into CAFs, which then undergo crosstalk with tumor cells, immune cells, and other stromal cells and ultimately lead to the active modification of the TME, resulting in an immunosuppressive state. Therefore, it is potentially valuable to develop biomarkers based on CCCT. However, few studies have incorporated the crosstalk between CAFs and tumor cells to guide prognosis and precision treatment strategies for HNSCC.

In this study, by using scRNA-seq data and bulk transcriptomic data of HNSCC patients, we developed the CCRGPI based on CCCT and performed multiple validations to assess its performance and reliability. Our results showed that patients in the high-CCRGPI group exhibited lower immune cell infiltration, lower immunogenicity, poorer prognosis, and poorer immunotherapeutic response than those in the low-CCRGPI group. These findings suggest that the CCRGPI may be a promising and reliable biomarker for predicting the prognosis and immunotherapy response of HNSCC patients. Moreover, although HPV-positive and HPV-negative HNSCC patients have different immune response patterns, the CCRGPI showed consistently performance in both HPV subgroups, which further confirmed the rationality of establishing a prognostic model based on CCCT.

Some studies have revealed that the higher the content of CAFs, the worse the prognosis of HNSCC patients [37, 51]; however, it has also been shown that the content of CAFs has no significant association with the prognosis of HPV-positive HNSCC patients [52]. This contradiction may be related to the different measurement methods and standards used. In addition, the identification of the content of CAFs in previous studies was mostly based on the expression of marker genes of CAFs. However, these marker genes are differentially expressed in the subpopulations of CAFs, and the contents of these subpopulations also vary in different HNSCC patients. This heterogeneity makes the measurement of the CAFs’ content prone to error, and the prognostic indicators based on the content of CAFs are also difficult to be accurate. In our study, we incorporated two major CAFs subpopulations (MatCAF and MyoCAF) into the model. Notably, there was no significant difference in the contents of the two CAFs subpopulations between the two CCRGPI subgroups. This reduces the impact of the content of each subpopulation on the predictive ability of the model, thus minimizing the error in the prediction. On the one hand, this enhances the reliability of our model; on the other hand, it also highlights the necessity of constructing a prognostic model based on the heterogeneity of the TME.

To identify potential small-molecule drugs for clinical combination therapy, we constructed a PPI network for the eight genes contained in the CCRGPI. In this network, we screened IGF1R as a key drug target. IGF1R is an oncogene that promotes tumor cell proliferation, metabolism, and metastasis [53], and it has promising applications in combination therapy for tumors [54]. By molecular docking technique, we found that Ganetespib has a high affinity with IGF1R. The anticancer effects of Ganetespib have been widely recognized [55, 56], and Ganetespib can inhibit IGF1R expression [57, 58]. Therefore, Ganetespib has the potential to be combined with cancer immunotherapy to improve the prognosis of HNSCC patients at high risk. However, further in-depth studies and experimental validation of Ganetespib and its specific molecular mechanisms are needed.

The construction of the prognostic model based on CCCT in this study has multiple advantages. On the one hand, the model incorporates the two main factors causing TME heterogeneity, CAFs and tumor cells, thus reducing the impact of TME heterogeneity on the prediction accuracy of the model. On the other hand, this study constructed pairLRs for the crosstalk between CAFs and tumor cell subpopulation at the single-cell level, thereby connecting CAFs and tumor cells into a whole, which is more in line with the actual situation in vivo. However, this study has several limitations. The CCRGPI was constructed using retrospective data obtained from public databases, which has a potential bias. Future validation of this index should focus more on prospective studies and need to incorporate multicenter HNSCC cohort data. In addition, few HNSCC immunotherapy cohorts with survival data are accessible at present, and CCRGPI needs to be validated in more HNSCC immunotherapy cohorts. Moreover, the molecular network that determines the function of CCRGPI during the development of HNSCC deserves further investigation.

## Conclusions

Overall, we developed a reliable CCCT-based prognostic index for HNSCC and explored potential small-molecule drugs for clinical combination therapy. It is hoped that the CCRGPI will be used for predicting prognosis and immunotherapy response and guiding the individualized treatment for HNSCC patients.

### Supplementary Information

**Additional file 1: Figure S1.** Analysis of the scRNA-seq data. (A) Quality control of the scRNA-seq data. (B) Integration of the scRNA-seq data. (C) Expression of marker genes of CAFs in three CAFs subgroups. (D) Highly expressed genes in single-cell subgroups. CAFs, cancer-associated fibroblasts. **Figure S2.** PairLRs involved in CCCT. CCCT, cancer-associated fibroblast (CAF)-cancer cell crosstalk; pairLRs, paired ligands and receptors. **Figure S3.** Construction and Validation of the CCRGPI. (A) PairLRs selected from LASSO regression analysis. (B) Proportion of MatCAF, MyoCAF, SCC1, and SCC2 cells in the TCGA cohort (ns: not significant, *p < 0.05). (C) Proportion of patients with various clinicopathologic factors in different CCRGPI subgroups. CCRGPI, cancer-associated fibroblast (CAF)-cancer cell crosstalk-related gene prognostic index; LASSO, least absolute shrinkage and selection operator; pairLRs, paired ligands and receptors; MatCAF, matrix CAF; MyoCAF, myofibroblast; SCC2, squamous cell carcinoma 2; TCGA, The Cancer Genome Atlas. **Figure S4.** Expression of pairLRs contained in the CCRGPI in scRNA-seq data. CCRGPI, cancer-associated fibroblast (CAF)-cancer cell crosstalk-related gene prognostic index; pairLRs, paired ligands and receptors. **Figure S5.** Differential expression analysis in different CCRGPI subgroups. (A) Volcano map showing differentially expressed genes. (B) Activated and inhibited HALLMARK pathways in the high-CCRGPI group. (C) The top 10 genes with the highest mutation frequencies in different CCRGPI subgroups. (D) Forest plot showing genes with mutational differences in different CCRGPI subgroups (**p < 0.01, ***p < 0.001). (E) Interaction effect of genes with significant mutation differences. (F) Interaction effect of genes contained in the CCRGPI (*p < 0.05,^**.**^p < 0.01). (G) TMB in different CCRGPI subgroups. CCRGPI, cancer-associated fibroblast (CAF)-cancer cell crosstalk-related gene prognostic index; TMB, tumor mutation burden. **Figure S6.** Immune characteristics in different HPV subgroups. (A) Correlation between the immunescore and CCRGPI in different HPV subgroups (ns: not significant, *p < 0.05). (B) Correlation between the ES of immune-related gene sets and CCRGPI in different HPV subgroups (*p < 0.05, **p < 0.01, ***p < 0.001). (C) Immune checkpoints differentially expressed among CCRGPI subgroups in different HPV subgroups (*p < 0.05, **p < 0.01, ***p < 0.001, ****p < 0.0001). (D) Proportion of immune cells based on the XCELL, TIMER, MCPCOUNTER, EPIC, and CIBERSORT algorithms in different HPV subgroups. CCRGPI, cancer-associated fibroblast (CAF)-cancer cell crosstalk-related gene prognostic index; ES, enrichment score; HPV, human papillomavirus. **Figure S7.** Screening of small-molecule drugs and molecular docking. (A) Schematic diagram of the CMap analysis. (B) PPI network of genes contained in the CCRGPI. (C) The three-dimensional interaction diagrams of Ganetespib with IGF1R. CCRGPI, cancer-associated fibroblast (CAF)-cancer cell crosstalk-related gene prognostic index; CMap, connectivity map; PPI, protein–protein interaction.

**Additional file 2: Table S1.** The Predicted IC50 values of the 83 drugs with statistical significance. **Table S2.** The results of molecular docking

## Data Availability

Data are available upon reasonable request.

## Abbreviations

AIC: Akaike information criterion
AUC: Area under the receiver operating characteristic curve
Ca-mg: Matrigel containing cancer cells
CCRGPI: Cancer-associated fibroblast (CAF)-cancer cell crosstalk-related gene prognostic index
CCCT: Cancer-associated fibroblast (CAF)-cancer cell crosstalk
CMap: Connectivity map
CTLA4: Cytotoxic T-lymphocyte-associated protein 4
DEGs: Differentially expressed genes
ES: Enrichment score
FCICC: Fibroblast-cancer cells indirect coculture
FC-organoid: Fibroblast-cancer cell organoid
Fib-mg: Matrigel containing fibroblasts
GEO: Gene Expression Omnibus
GO: Gene Ontology
GSEA: Gene set enrichment analysis
HNSCC: Head and neck squamous cell carcinoma
HPV: Human papillomavirus
IC50: 50% Inhibitory concentration
ICI: Immune checkpoint inhibitor
IPS: Immunophenoscore
IRGPI: Immune-related gene prognostic index
IRGS: Immune-related gene signature
LASSO: Least absolute shrinkage and selection operator
LTS: Long-term survivors
m6Arisk: M6A-based risk score
MatCAF: Matrix CAF
MyoCAF: Myofibroblast
OS: Overall survival
pairLRs: Paired ligands and receptors
PCA: Principal component analysis
PCs: Principal components
PD-1: Programmed death 1
PDB: Protein Data Bank
PPI: Protein–protein interaction
Resfib: Resting fibroblast
SCC: Squamous cell carcinoma
ssGSEA: Single sample gene set enrichment analysis
STS: Short-term survivors
TCGA: The Cancer Genome Atlas
TCIA: The Cancer Immunome Database
TIDE: Tumor immune dysfunction and exclusion
TMB: Tumor mutation burden
TME: Tumor microenvironment
