# Supplementary figures and images for: A novel CAF-cancer cell crosstalk-related gene prognostic index based on machine learning: prognostic significance and prediction of therapeutic response in head and neck squamous cell carcinoma
Source: J Transl Med. 2024 Jul 9;22:645. doi: 10.1186/s12967-024-05447-6 (PMC11234636; doi:10.1186/s12967-024-05447-6)

Figure S1

A

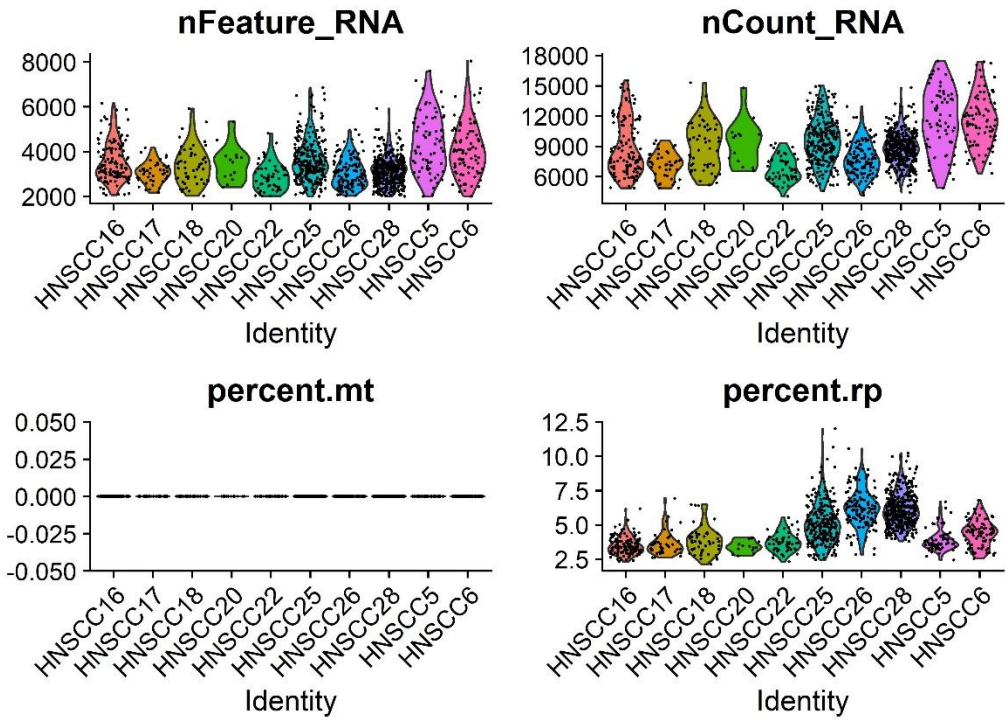

B

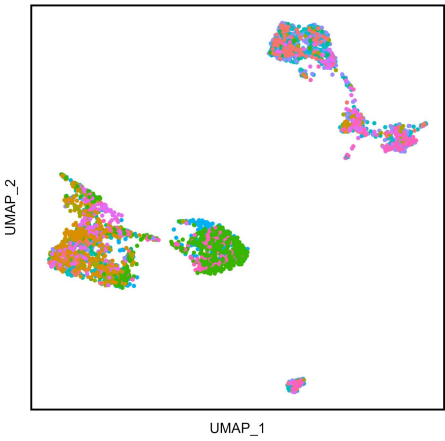

C

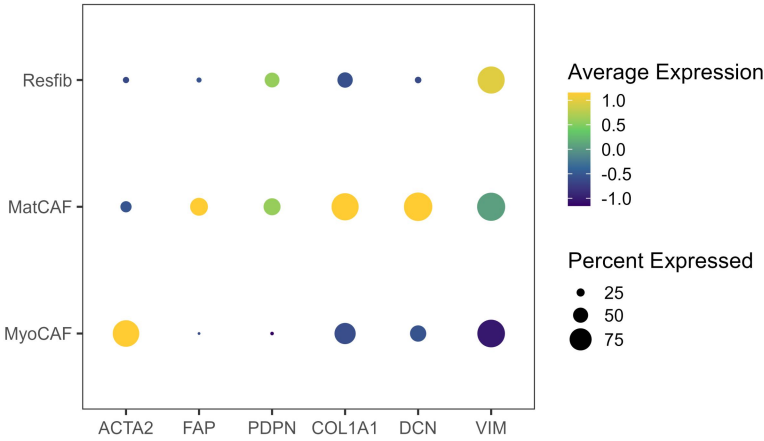

D

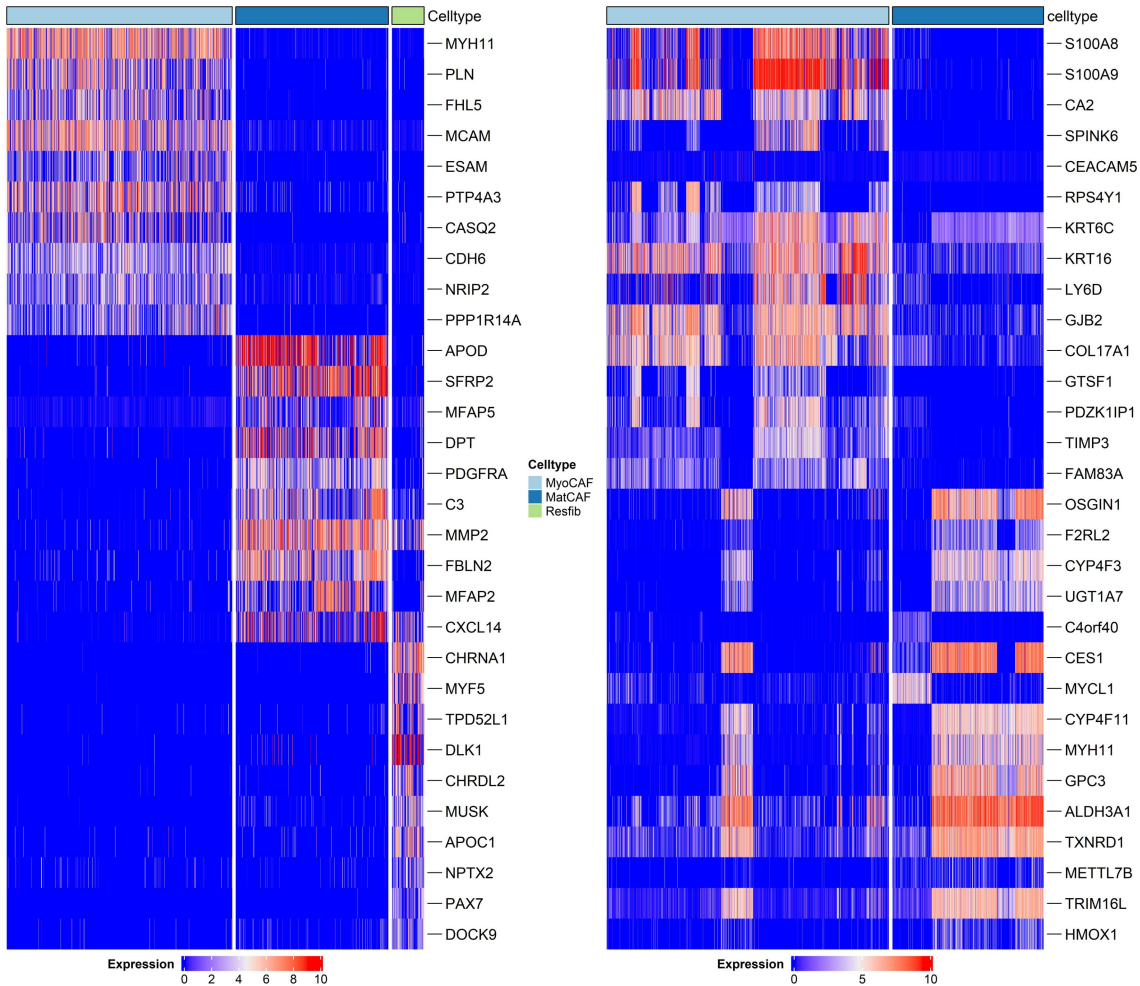

Figure S2

A

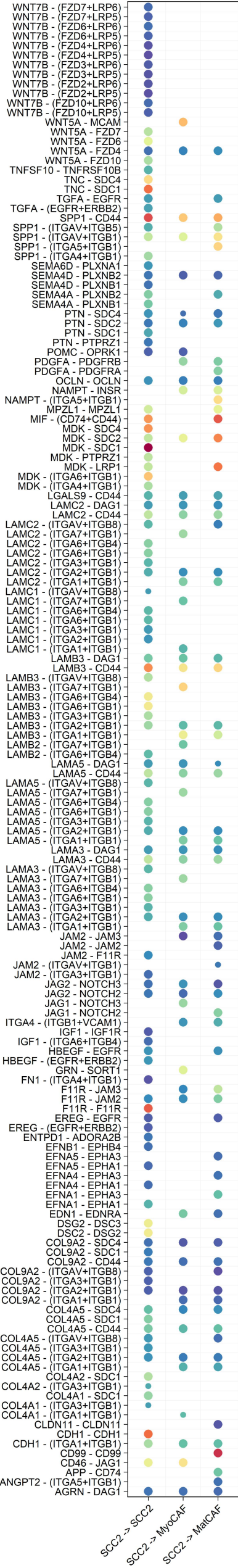

B

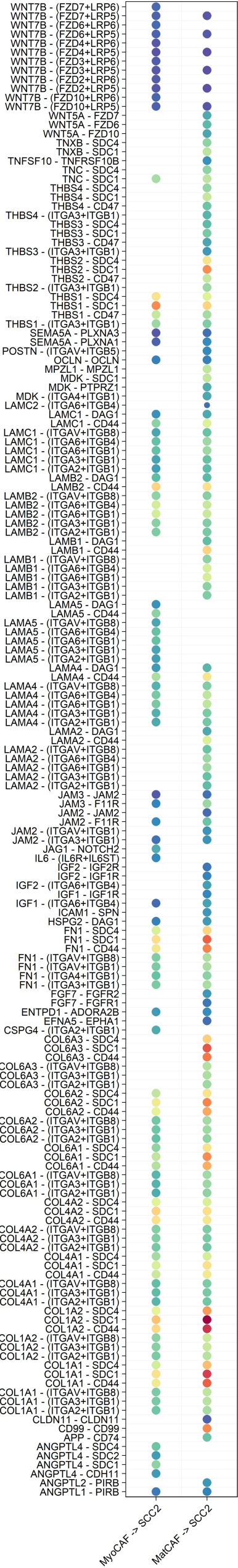

Figure S3

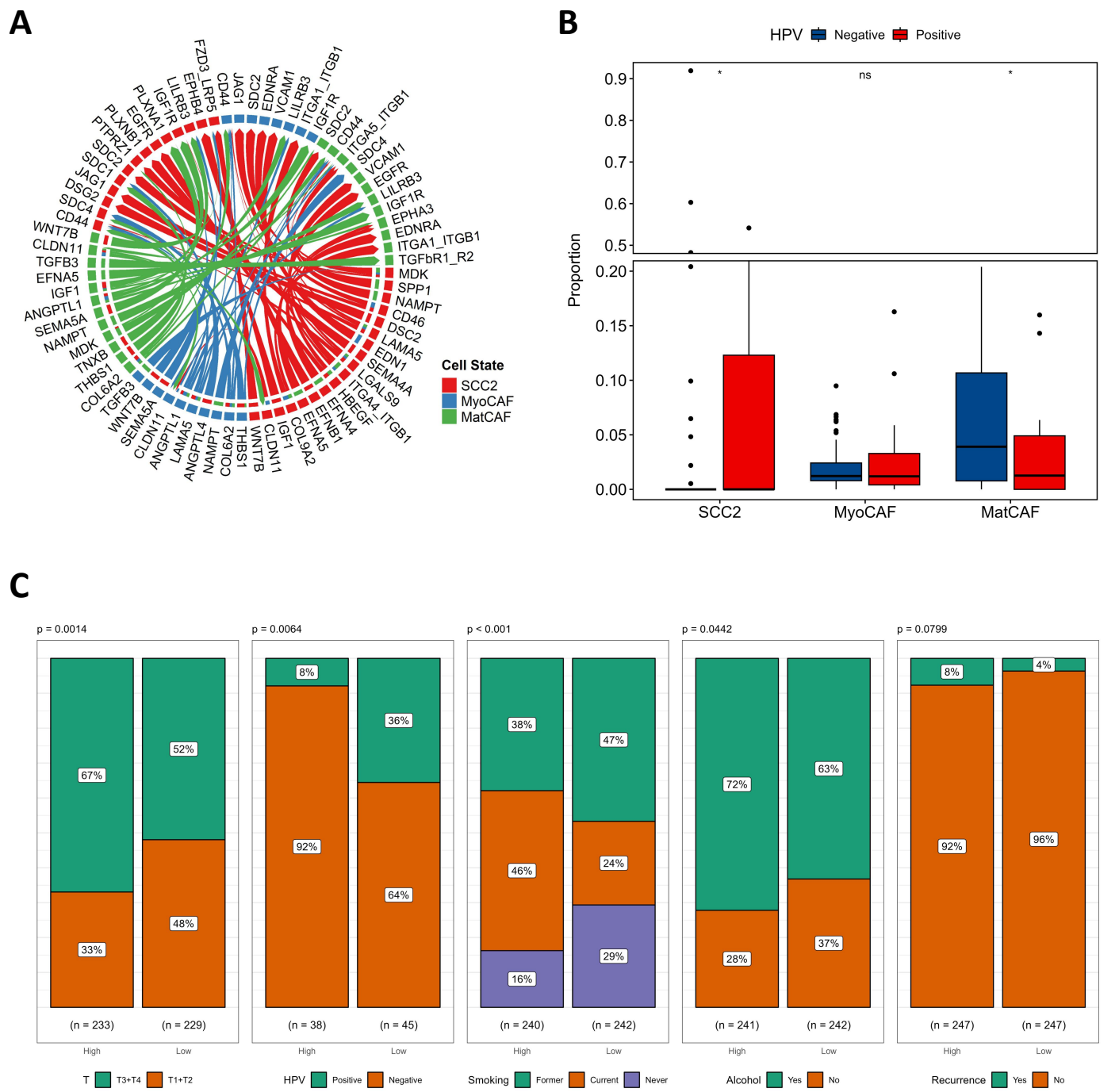

Figure S4

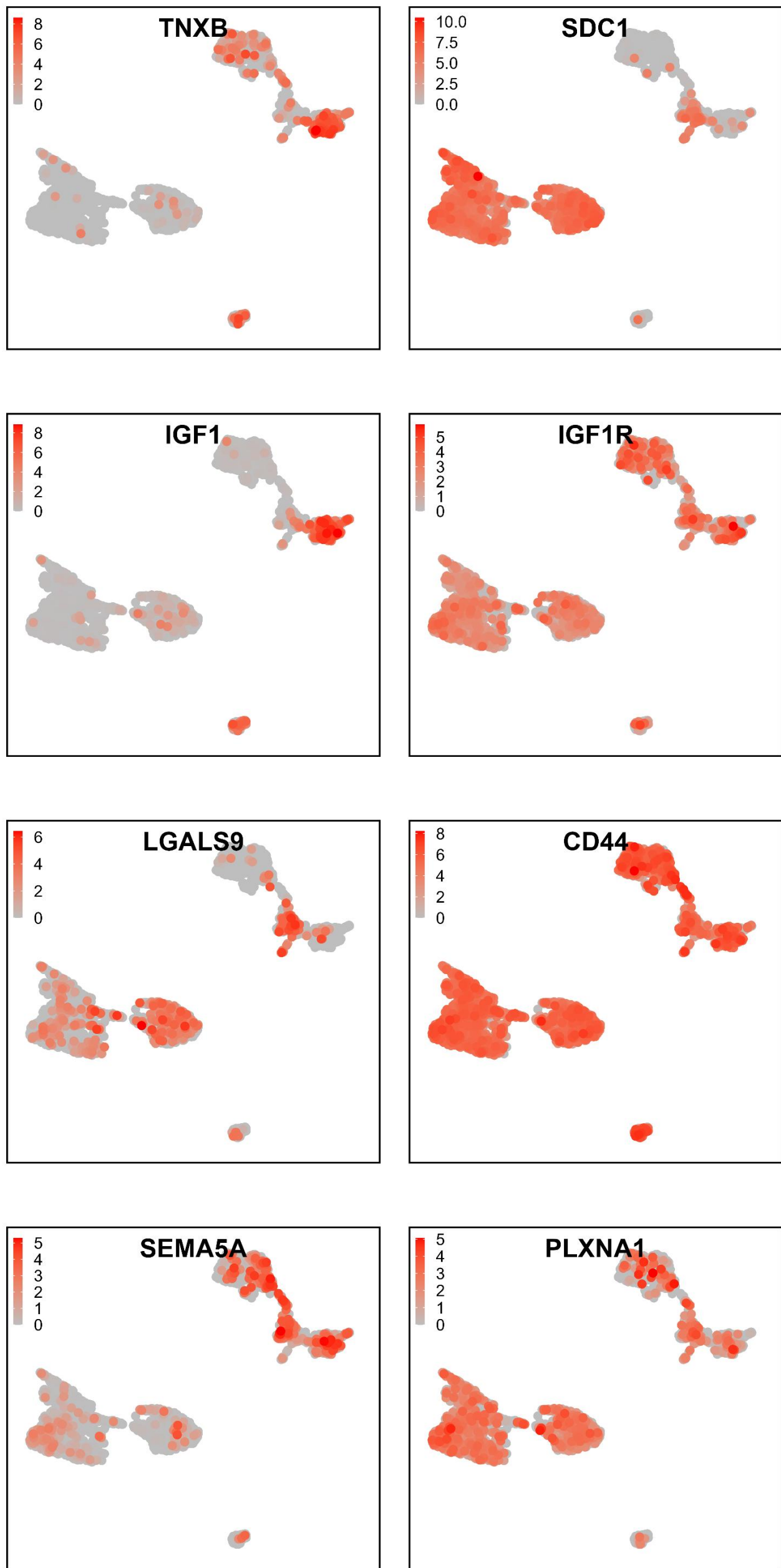

Figure S5

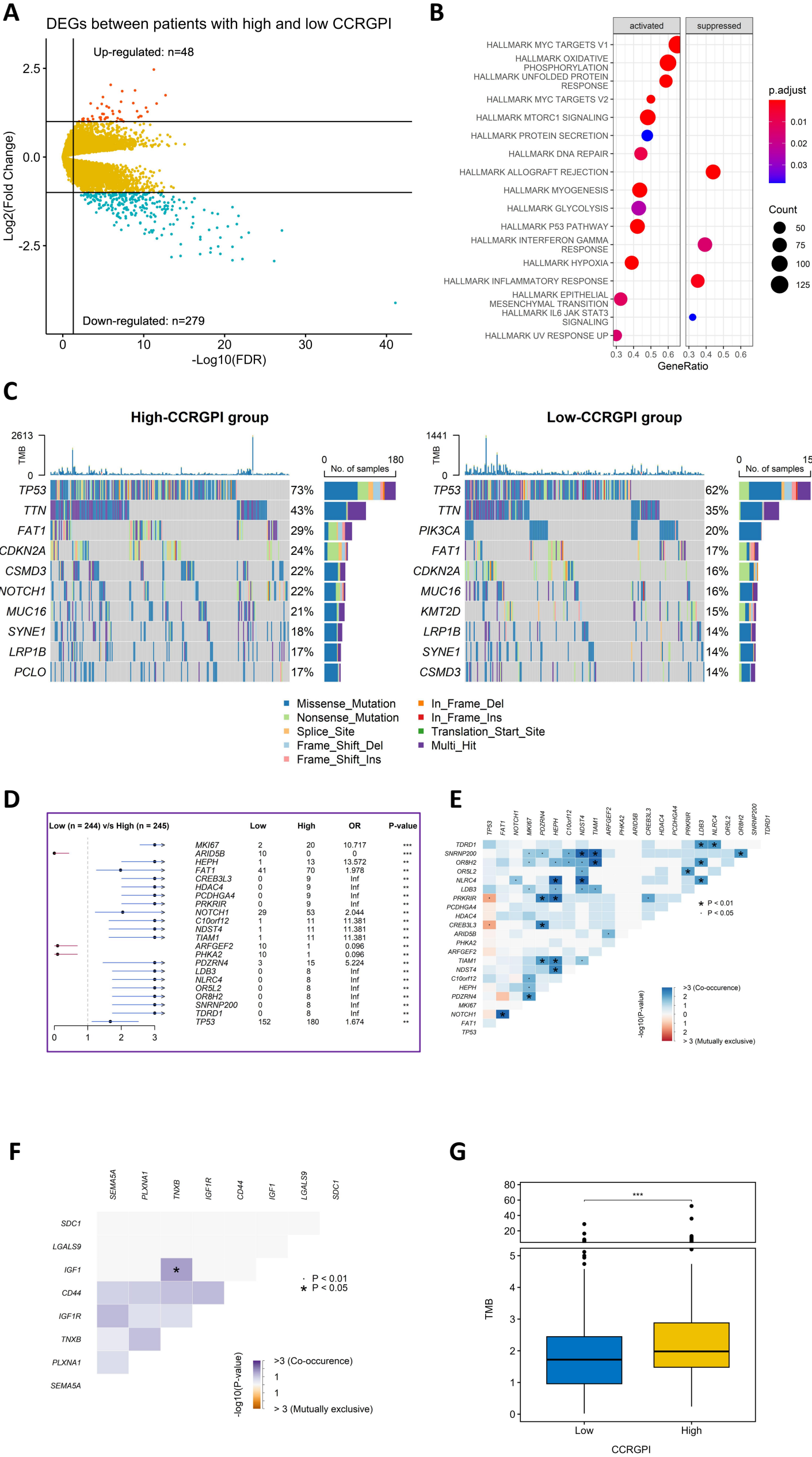

Figure S6

A

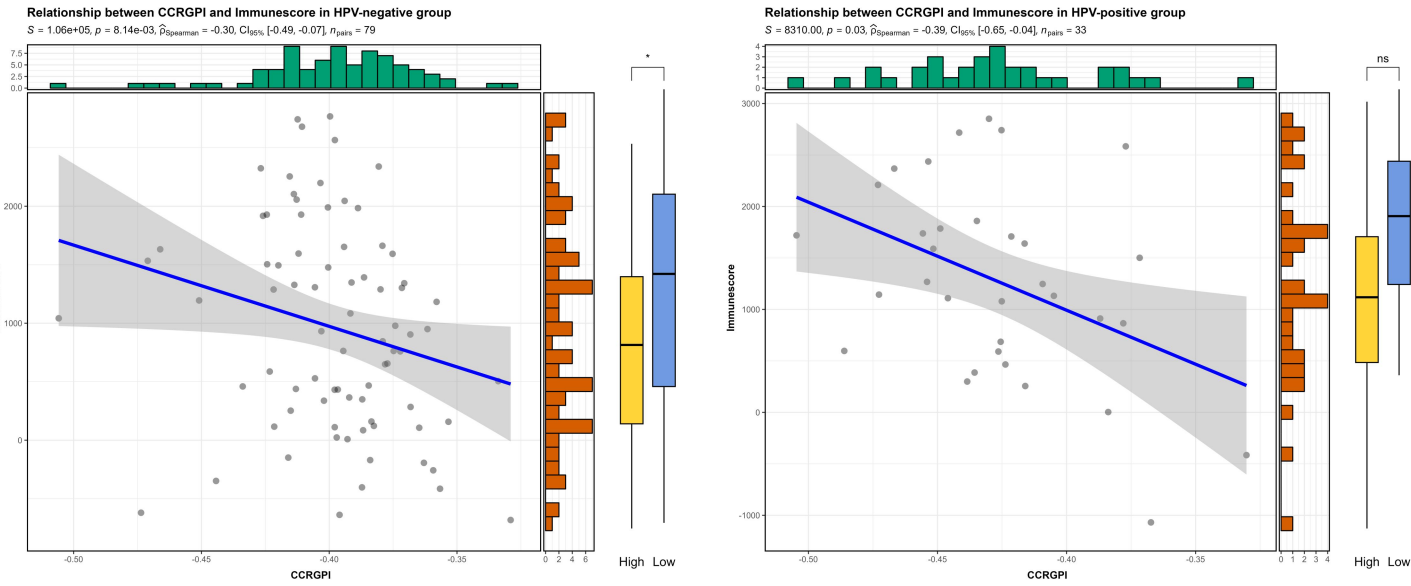

B

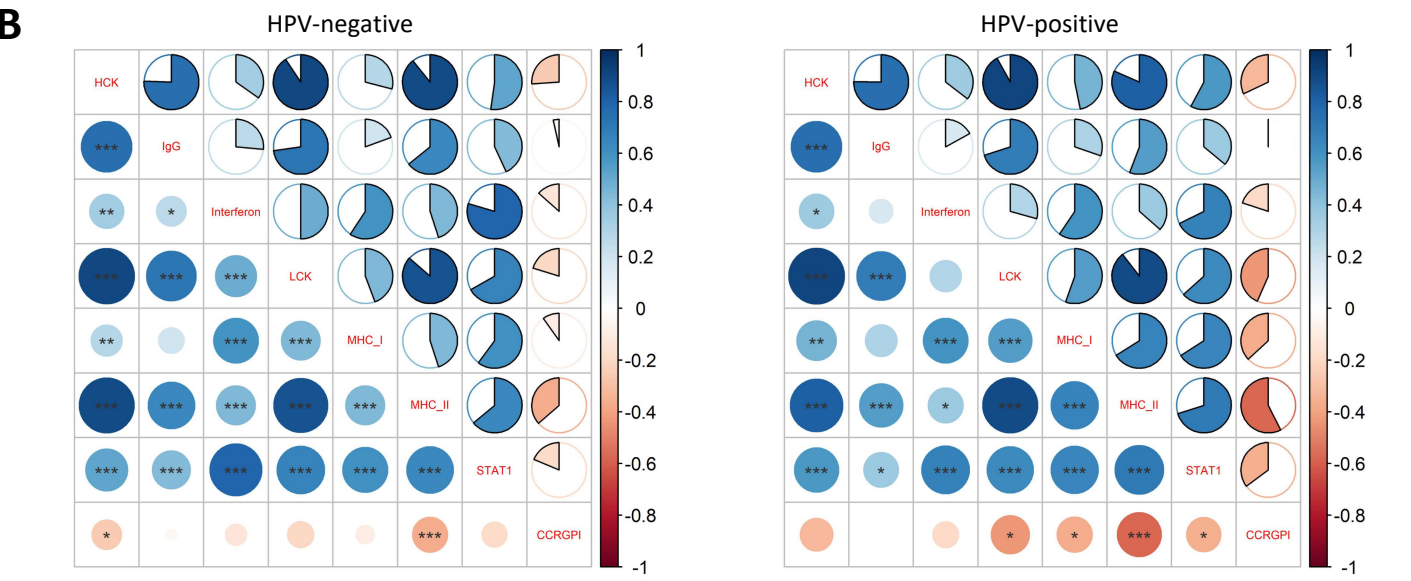

C

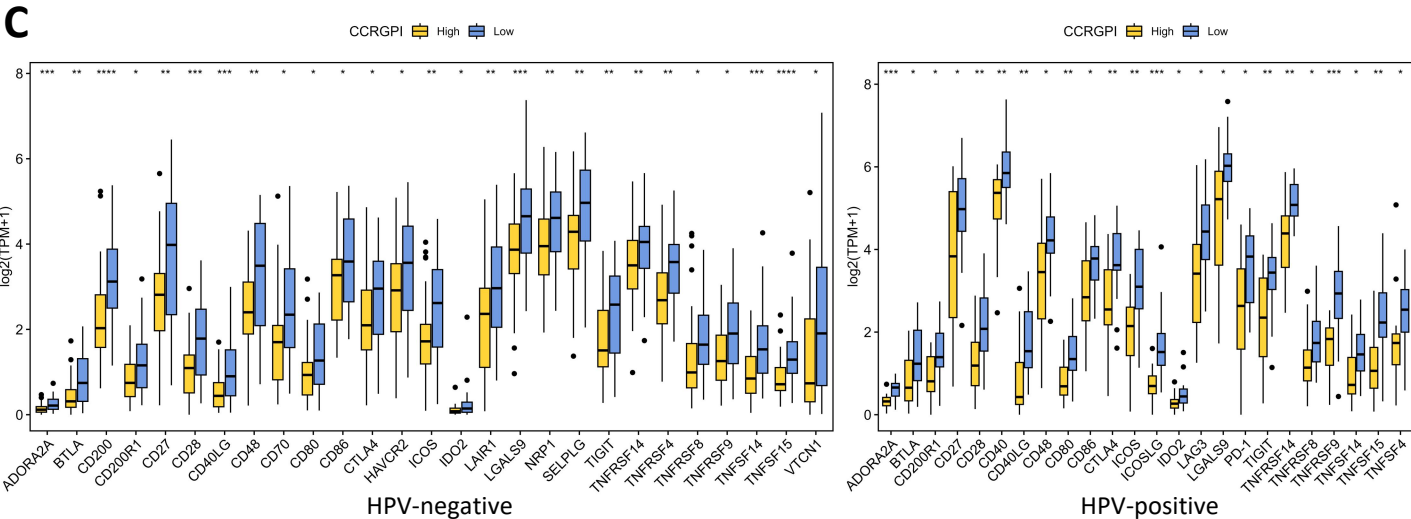

D

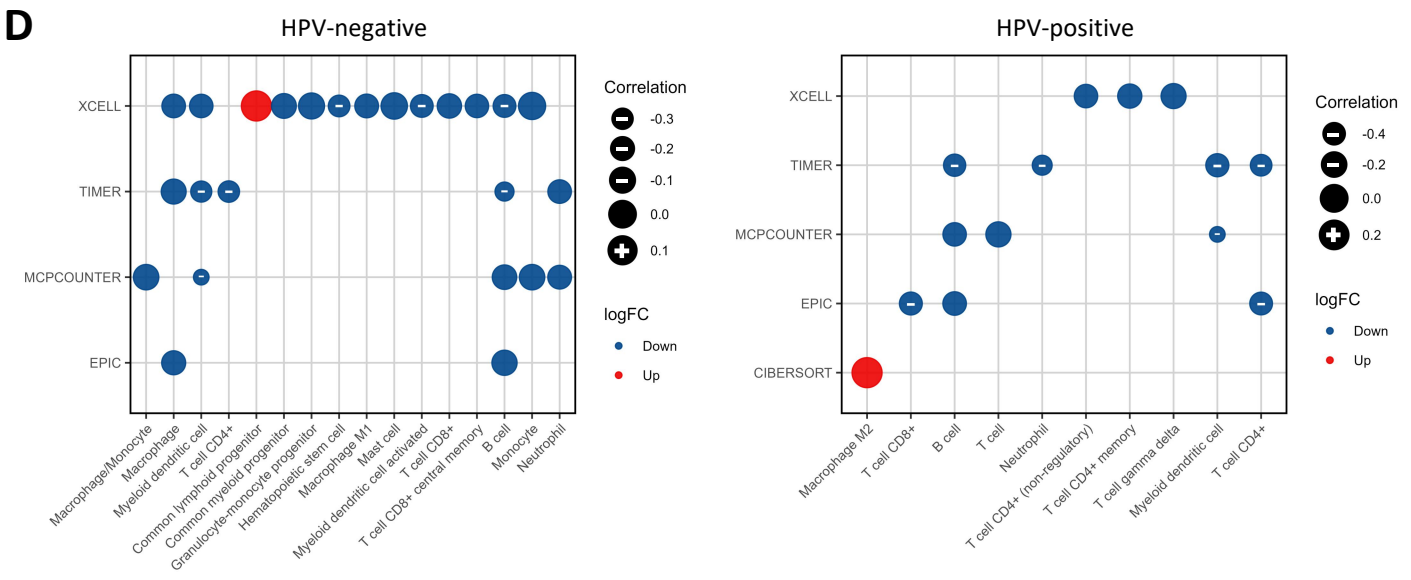

Figure S7

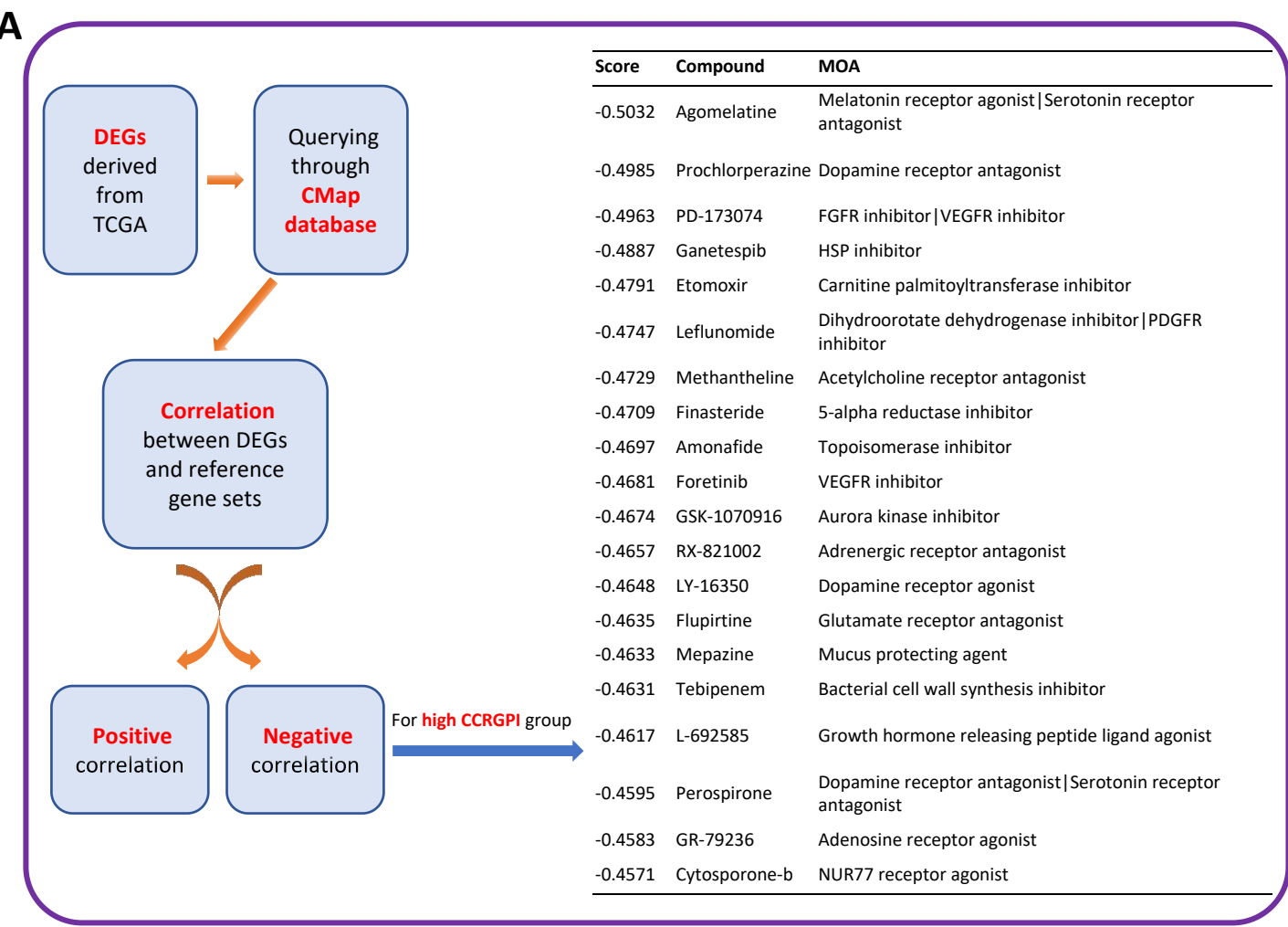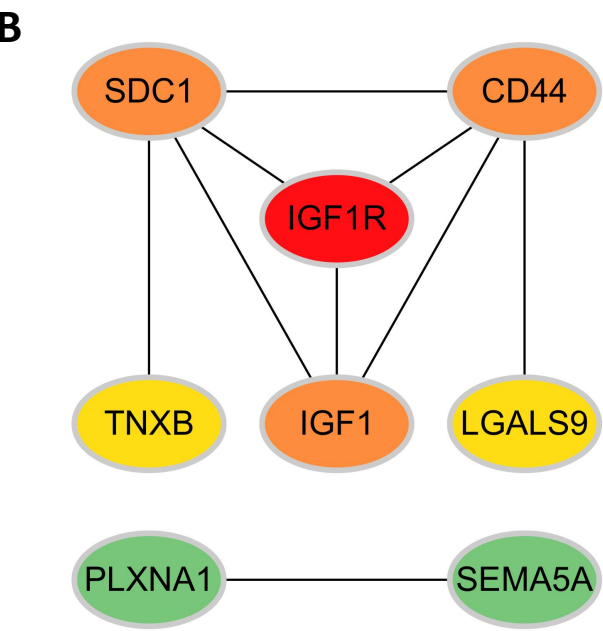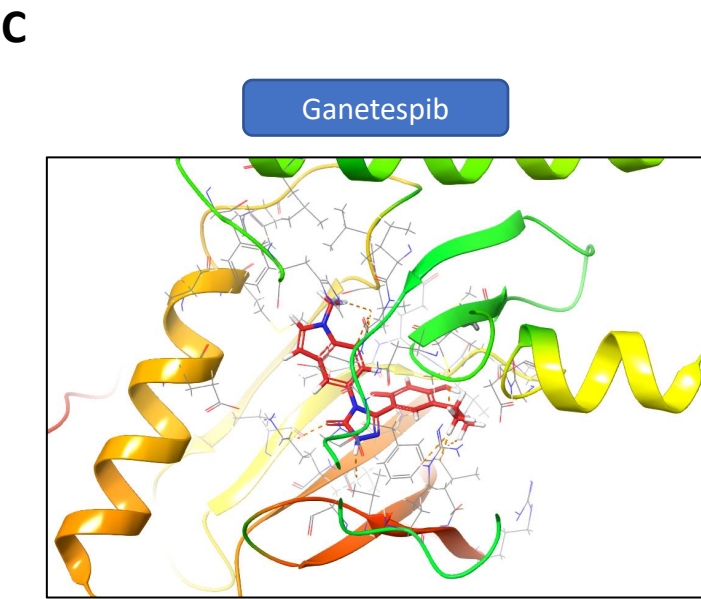

Supplement: Supplementary file 1 — Additional file 1: Figure S1. Analysis of the scRNA-seq data. (A) Quality control of the scRNA-seq data. (B) Integration of the scRNA-seq data. (C) Expression of marker genes of CAFs in three CAFs subgroups. (D) Highly expressed genes in single-cell subgroups. CAFs, cancer-associated fibroblasts. Figure S2. PairLRs involved in CCCT. CCCT, cancer-associated fibroblast (CAF)-cancer cell crosstalk; pairLRs, paired ligands and receptors. Figure S3. Construction and Validation of the CCRGPI. (A) PairLRs selected from LASSO regression analysis. (B) Proportion of MatCAF, MyoCAF, SCC1, and SCC2 cells in the TCGA cohort (ns: not significant, *p < 0.05). (C) Proportion of patients with various clinicopathologic factors in different CCRGPI subgroups. CCRGPI, cancer-associated fibroblast (CAF)-cancer cell crosstalk-related gene prognostic index; LASSO, least absolute shrinkage and selection operator; pairLRs, paired ligands and receptors; MatCAF, matrix CAF; MyoCAF, myofibroblast; SCC2, squamous cell carcinoma 2; TCGA, The Cancer Genome Atlas. Figure S4. Expression of pairLRs contained in the CCRGPI in scRNA-seq data. CCRGPI, cancer-associated fibroblast (CAF)-cancer cell crosstalk-related gene prognostic index; pairLRs, paired ligands and receptors. Figure S5. Differential expression analysis in different CCRGPI subgroups. (A) Volcano map showing differentially expressed genes. (B) Activated and inhibited HALLMARK pathways in the high-CCRGPI group. (C) The top 10 genes with the highest mutation frequencies in different CCRGPI subgroups. (D) Forest plot showing genes with mutational differences in different CCRGPI subgroups (**p < 0.01, ***p < 0.001). (E) Interaction effect of genes with significant mutation differences. (F) Interaction effect of genes contained in the CCRGPI (*p < 0.05,.p < 0.01). (G) TMB in different CCRGPI subgroups. CCRGPI, cancer-associated fibroblast (CAF)-cancer cell crosstalk-related gene prognostic index; TMB, tumor mutation burden. Figure S6. Immune c [file 12967_2024_5447_MOESM1_ESM.pdf]
